# Health Dataspace Technology Acceptance Model (HDS-TAM): Development and Case Evaluation Study of Patient Acceptance of Germany’s Electronic Patient Record

**DOI:** 10.2196/99366

**Published:** 2026-11-02

**Authors:** Veronika Vasileva, Eduard Wolf, Volker Riediger, Amir Shayan Ahmadian, Daniel Buschmann, Jan Jürjens

**Affiliations:** 1 Institute for Software Technology, Faculty of Computer Science, Universität Koblenz, Universitätsstr. 1, Koblenz, Rheinland-Pfalz, 56070, Germany, 49 2612870; 2 Healthcare, Fraunhofer Institute for Software and Systems Engineering, Dortmund, North Rhine-Westphalia, Germany

**Keywords:** health dataspace, electronic health records, technology acceptance, transparency, elektronische patientenakte, ePA, German electronic patient record

## Abstract

**Background:**

The European Health Dataspace (EHDS) expands health data sharing beyond direct care and introduces governance, transparency, trust, and secondary-use considerations that are not fully captured by established technology acceptance models such as the Technology Acceptance Model (TAM) and the Unified Theory of Acceptance and Use of Technology (UTAUT). To address this gap, we developed the health dataspace technology acceptance model (HDS-TAM) and evaluated it using the German electronic patient record (ePA) as an illustrative health dataspace (HDS) application.

**Objective:**

This study aimed (1) to derive an HDS-oriented technology acceptance framework from the literature and (2) to assess its applicability in explaining acceptance of the ePA from a patient perspective.

**Methods:**

A structured literature review screened 1788 records, reviewed 134 full-text papers, and retained 68 studies for model development. HDS-TAM was subsequently applied in a scenario-based online survey conducted between March and April 2025. Of 235 questionnaire accesses and 163 submitted responses, 149 valid responses were analyzed. Data were analyzed using multiple imputation, exploratory factor analysis, and partial least squares structural equation modeling (PLS-SEM). The association between behavioral intention and actual ePA use was examined using logistic regression.

**Results:**

The model explained 75.9% of the variance in behavioral intention to use (BI, *R*²=0.759), 67% in perceived usefulness (PU, *R*²=0.670), and 54% in perceived risk (PR, *R*²=0.540). PU showed the strongest positive association with BI (*β*=.670, *P*<.001, *f*²=0.457), whereas PR showed a small negative association (*β*=−.149, *P*=.04, *f*²=0.034). Transparency was positively associated with PU (*β*=.220, *P*=.001) and perceived ease of use (PEOU; *β*=.454, *P*<.001) and negatively associated with PR (*β*=−.594, *P*<.001). Perceived need (PN) showed a strong positive association with PU (*β*=.567, *P*<.001) but no significant direct association with BI (*β*=.071, *P*=.35). Eight of 20 hypotheses were supported. Only 23.5% (n=35) of participants reported actual ePA use. BI showed a positive but imprecise and nonsignificant association with self-reported actual use (OR 1.37, 95% CI 0.89-2.11, *P*=.15). Measurement quality was mixed: reflective constructs generally showed satisfactory reliability and convergent validity; however, discriminant validity between PU and BI was poor (heterotrait-monotrait ratio of correlations [HTMT]=0.981).

**Conclusions:**

PU showed the strongest association with behavioral intention in the ePA case evaluation, while transparency and PN were mainly associated with behavioral intention through indirect pathways. The findings suggest potential value in extending traditional acceptance models with governance-related constructs relevant to HDSs. However, the severe overlap between PU and behavioral intention, low actual ePA uptake, and the young, highly educated convenience sample limit confidence in the magnitude and distinctiveness of the observed effects. HDS-TAM should therefore be regarded as a promising but preliminary framework requiring further refinement and validation.

## Introduction

### Background and Motivation

The European Health Dataspace (EHDS) regulation establishes a common framework for cross-border access and exchange of electronic health data for primary use (health care delivery) and regulated secondary use (research, innovation, or policy-making) under defined governance conditions [1]. EHDS strengthens individuals’ rights to access and control their electronic health data, including mechanisms to restrict health professionals’ access [2].

A *dataspace* is a distributed system governed by a framework that enables secure, trustworthy data transactions while supporting data sovereignty [3]. A health dataspace (HDS) is a sector-specific dataspace where multiple participants (eg, patients, health care providers, or insurers) share, access, and reuse electronic health data through federated governance and enabling infrastructures while preserving data sovereignty and ensuring trustworthy transactions. HDSs enable data-driven quality improvement, research, and innovation, while reducing duplication and supporting safer clinical decision-making. These depend not only on technical interoperability and organizational deployment but also on public trust, patient participation, and perceived legitimacy of data sharing.

A HDS challenges classic assumptions of acceptance research. Rather than operating the underlying infrastructure, patients frequently act as data participants affected by cross-organizational data flows and governance decisions. Acceptance in this context includes not only the intention to use a specific system or application, but also the perceived legitimacy of data sharing, access governance, control options, and secondary use. Consequently, acceptance is multifaceted: individuals may accept digitization and sharing for direct care (primary use), while remaining cautious about reuse beyond care (secondary use) [4].

Empirical syntheses indicate that people are more comfortable with data sharing when it is clearly tied to their own care and more cautious regarding secondary use, particularly when transparency, governance safeguards, or trust in institutions is limited [4,5]. Willingness to share depends on perceived public benefit, the trustworthiness of data handlers, privacy and security expectations, and perceived control or oversight [6]. Research on social acceptance of data-intensive health care suggests that legal compliance alone is insufficient. Durable acceptance requires visible trust-building, public value articulation, and governance practices viewed as fair and accountable [7].

Despite decades of electronic health record rollouts and health care innovation and education initiatives, concerns about privacy, misuse, opaque data flows, and unequal benefits can persist and undermine participation and legitimacy, particularly as infrastructures scale from organizational systems to cross-border dataspaces. Patient and public perspectives are crucial, as they influence behaviors such as restricting access, withholding information, or opting out, thereby affecting the feasibility of primary and secondary use at scale. This motivates an integrated framework for assessing multistakeholder acceptance tailored to the concept of HDSs.

Although established frameworks such as the Technology Acceptance Model (TAM) [8] and the Unified Theory of Acceptance and Use of Technology (UTAUT) [9] offer foundational constructs (eg, usefulness and effort expectations), they cannot capture the key characteristics of HDSs without major adaptation [8-10]. Traditional TAM-based approaches were developed for contexts with clear end users and direct system interaction. Systematic reviews show that most TAM studies in health technologies extend the original model with contextual variables and conclude that no optimal version exists for health services [10]. They further highlight inconsistent findings and the need for context-specific adaptation [11]. UTAUT and its variants face similar issues regarding generalizability and model stability [12]. Health care–specific models such as the health care technology acceptance model (H-TAM) address narrowly defined populations and technologies and thus do not cover cross-stakeholder, governance-heavy dataspace settings [13].

These limitations highlight the need for an HDS-oriented acceptance framework, integrating governance, trust, control, and perceived public value alongside established technology acceptance constructs.

### Acceptance of HDSs and Health Data Sharing

We consider the acceptance of HDS as technology acceptance. This is defined as “the positive phenomenon of adopting an innovation by its users” [14]. Thus, the investigation of inhibiting and facilitating factors is important [9].

Research on the acceptance of HDSs and health data sharing is currently divided into three partly connected areas: (1) technical and governance-oriented work on dataspaces and the implementation of the EHDS; (2) empirical studies on public and patient willingness to share health data, especially for secondary use; and (3) technology acceptance research in digital health focusing on bounded technologies (eg, portals, electronic health record modules, and mHealth [mobile health] tools).

### Technical and Governance Perspectives

This area conceptualizes an HDS as a federated sociotechnical arrangement, focusing on architecture, interoperability, and governance (eg, access control and privacy or security), as well as implementation challenges—especially for secondary use [15,16]. However, it does not operationalize acceptance as an empirical construct nor provide a validated acceptance model for an HDS.

### Empirical Studies

A large evidence base examines willingness to share health data for research and other secondary purposes. Reviews and meta-analyses emphasize perceived public benefit, trust in data handlers, perceived control or choice, transparency, and concerns about misuse or commercial use [5,17]. While highly relevant for EHDS-style secondary use, they are commonly framed as attitudes toward data sharing rather than acceptance of systems and governance arrangements.

### Technology Acceptance Research

Health care acceptance studies widely apply TAM/UTAUT and variants to specific technologies; reviews highlight frequent model extensions and heterogeneous results [10,11,18]. Meta-analytic work further questions the stability and generalizability of UTAUT [12]. For HDSs, the main limitation is that governance and secondary-use concerns are treated as context-specific additions rather than integrated into an HDS acceptance model [10,19].

### Related Work on Technology Acceptance in Health Care

Several theories have been developed to explain technology acceptance, most notably TAM [8,20], later extended to TAM2 (Technology Acceptance Model version 2) [21] and TAM3 (Technology Acceptance Model version 3) [22]. Another widely used framework is UTAUT [9], which was further developed into the Unified Theory of Acceptance and Use of Technology version 2 (UTAUT2) [23] and later extensions with additional factors and moderators [12].

Although TAM and UTAUT are the most frequently applied models in health care, their explanatory power for behavioral intention is often limited. Studies extending UTAUT2 in health contexts report explained variance in behavioral intention ranging from 19.4% to 56% [24], suggesting that generic models may benefit from domain-specific extensions.

Prior studies also show inconsistent findings for individual determinants. For example, hedonic motivation was found to be insignificant in some studies [24] but highly relevant in others [25], indicating that acceptance depends on context and sample characteristics. Safi et al [26] further showed that patients with acute illnesses or severe prior disease experiences are more willing to adopt new technologies, suggesting that health status may shape acceptance.

To address the limitations of general models, the H-TAM was proposed for health self-management applications among older adults with hypertension [13]. It includes perceived ease of use (PEOU), perceived usefulness (PU), facilitating conditions, and social influence (SI) as determinants of behavioral intention. However, its empirical basis is limited to a small sample of 23 participants and a narrow application context, which restricts its generalizability to broader stakeholder groups and more complex systems such as HDSs. More broadly, Rahimi et al [10] identified a wide range of acceptance variables across 134 studies, varying by application scenario and stakeholder group.

Existing research therefore provides important foundations for understanding health technology acceptance and attitudes toward health data sharing, but it does not yet offer a reusable framework that integrates established acceptance constructs with HDS-specific concerns such as governance, trust, control, transparency, and public value. This gap is particularly relevant in HDS contexts, where patients are not only system users but also data participants affected by cross-organizational data flows and rules of access and reuse.

### Research Questions and Objectives

This paper develops the “health dataspace technology acceptance model (HDS-TAM)” as an HDS-oriented acceptance framework and applies it in a patient-focused case evaluation of the German electronic patient record (ePA). The ePA was selected as an illustrative HDS application because it combines direct patient-facing functionality with broader questions of data sharing, access control, and secondary use. The study addresses the following research questions (RQs):

- RQ1. How can an HDS-oriented acceptance framework be derived from established technology acceptance models and HDS-related concerns?
- RQ2. How does the proposed HDS-TAM perform in a patient-focused case evaluation of the ePA?

To answer these questions, we first derive HDS-TAM from a structured review of relevant literature and then operationalize selected constructs in a scenario-based survey assessing the ePA from a patient perspective. The empirical study is intended as an application and plausibility test of the framework, as well as a first indication of which factors are particularly relevant for patient acceptance in this context.

## Methods

### Research Approach

This study followed a 2-step research approach comprising framework development and case evaluation. In step 1, we conducted a structured literature review to identify constructs, variables, and influence relationships relevant to technology acceptance in HDSs. These findings were used to derive the HDS-TAM. In step 2, we conducted a patient-focused case evaluation using the ePA as an illustrative HDS technology.

The ePA was selected because it represents a real-world digital health application that combines direct patient use with broader questions of data access, control, governance, and secondary use. The case evaluation comprised 4 activities: adaptation of HDS-TAM to the ePA context, development of a scenario-based questionnaire and hypotheses model, execution of an online survey, and statistical analysis of the resulting data. Figure 1 summarizes the overall study design.

**Figure 1.**
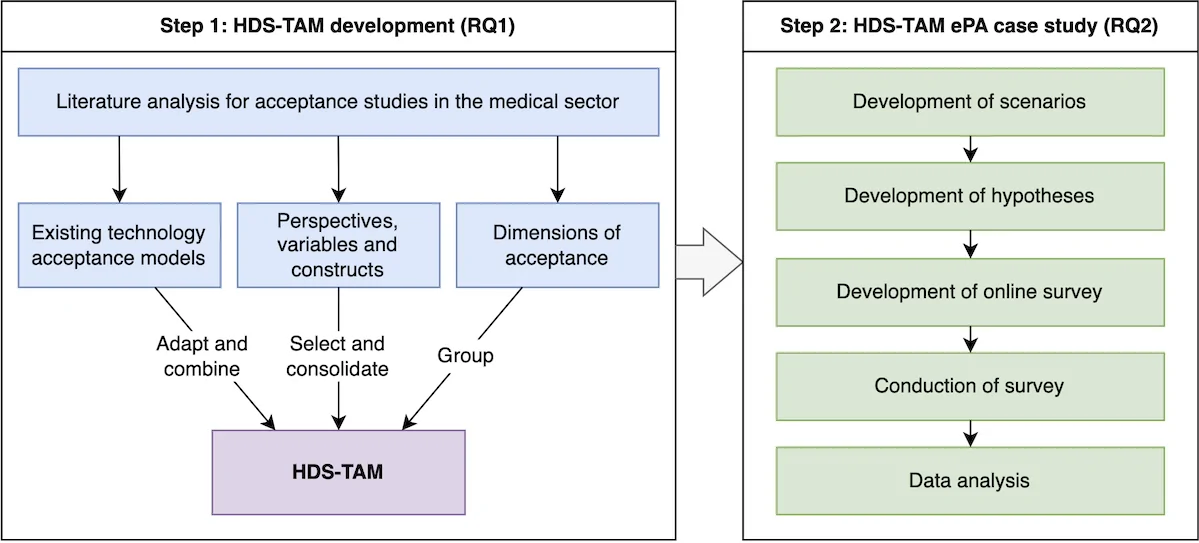
The research approach comprises 2 main steps: the development of HDS-TAM and its application to the ePA as a case evaluation. ePA: German electronic patient record; HDS-TAM: health dataspace technology acceptance model; RQ: research question.

### Ethical Considerations

The study consisted of an anonymous, noninterventional online survey and did not involve any clinical intervention or collection of directly identifying information. Under the institutional framework of the University of Koblenz, recourse to the university’s Commission for Ethics and Dual-Use Research is voluntary and takes place upon written application; the university’s regulations on good scientific practice require ethics approvals to be obtained where such approval is required [27,28]. Based on the anonymous, noninterventional, and minimal-risk nature of the study, the authors determined that formal review by the institutional ethics commission was not required and therefore did not seek an ethics review board assessment.

Before participation, respondents received information about the purpose and duration of the study, the voluntary nature of participation, and the anonymous handling of their responses. Informed consent was obtained before the survey commenced. Participants could discontinue participation or skip individual questions at any time without providing a reason. No compensation was provided. No directly identifying information was collected, and results are reported only in aggregate form.

### Step 1: HDS-TAM Development

#### Literature Review

To identify acceptance-related factors relevant to HDSs, we conducted a structured literature review following Kitchenham’s review guidance [29]. The search covered IEEE Xplore, Google Scholar, ScienceDirect, SpringerLink, Web of Science, PubMed, EBSCO Host, ACM, and ERIC. Search terms combined concepts related to HDSs and EHDS with terms from technology acceptance research, including data sharing, data governance, public value, transparency, technology acceptance, and UTAUT.

Studies were included if they addressed dataspaces or EHDS, explicitly examined health technology acceptance, or discussed barriers and challenges at the intersection of health data sharing and technology acceptance. Studies were excluded if they were not peer-reviewed, not written in English, or not related to health technologies or applications. After screening 1788 records, 134 publications were reviewed in full text, and 68 were retained for model development. From these studies, we extracted variables, constructs, and reported influence relationships relevant to acceptance in HDS-related contexts.

#### Model Building

Based on the reviewed literature, we developed HDS-TAM as an integrated acceptance framework for HDSs. Identified determinants were grouped into five perspectives: identity, aspects of technology, application environment, extrinsic motivators, and SIs. Identity includes user-related characteristics that shape acceptance, such as interests, personal innovativeness, experience, habit, education, technological affinity, and health condition. Aspects of technology cover system-related characteristics relevant to design and development. It includes established variables such as output quality, objective usability, performance expectancy, result demonstrability, hedonic motivation, familiarity, and complexity, as well as the additional HDS-specific variables: personalization, privacy, availability, transparency, and trust. The application environment includes context-related variables such as job relevance and compatibility, which reflect how well technology fits the domain in which it is used. Extrinsic motivators comprise externally shaped factors such as rewards or sanctions and facilitating conditions. These may include legal frameworks, financial incentives, or institutional support that encourage technology adoption. SIs include variables such as image, subjective norm, and perceived external influence, which capture the role of social expectations and public perception in acceptance. In addition, HDS-TAM introduces cross-cutting factor support and training, which may be provided by technology providers, health care institutions, or other external actors. These perspectives were further assigned to the 3 dimensions: acceptance subject (internal stimuli), acceptance object (external stimuli), and acceptance context (external stimuli).

Constructs and relationships from established models, particularly TAM3 [22], extended UTAUT [24], and H-TAM [13], were retained where relevant. Additional variables were added when prior literature suggested their relevance for HDS settings, especially in relation to governance, trust, privacy, transparency, control, and health-related need. Moderators from prior models were also retained, with the role generalized to account for different HDS stakeholders. The resulting model is shown in Figure 2.

**Figure 2.**
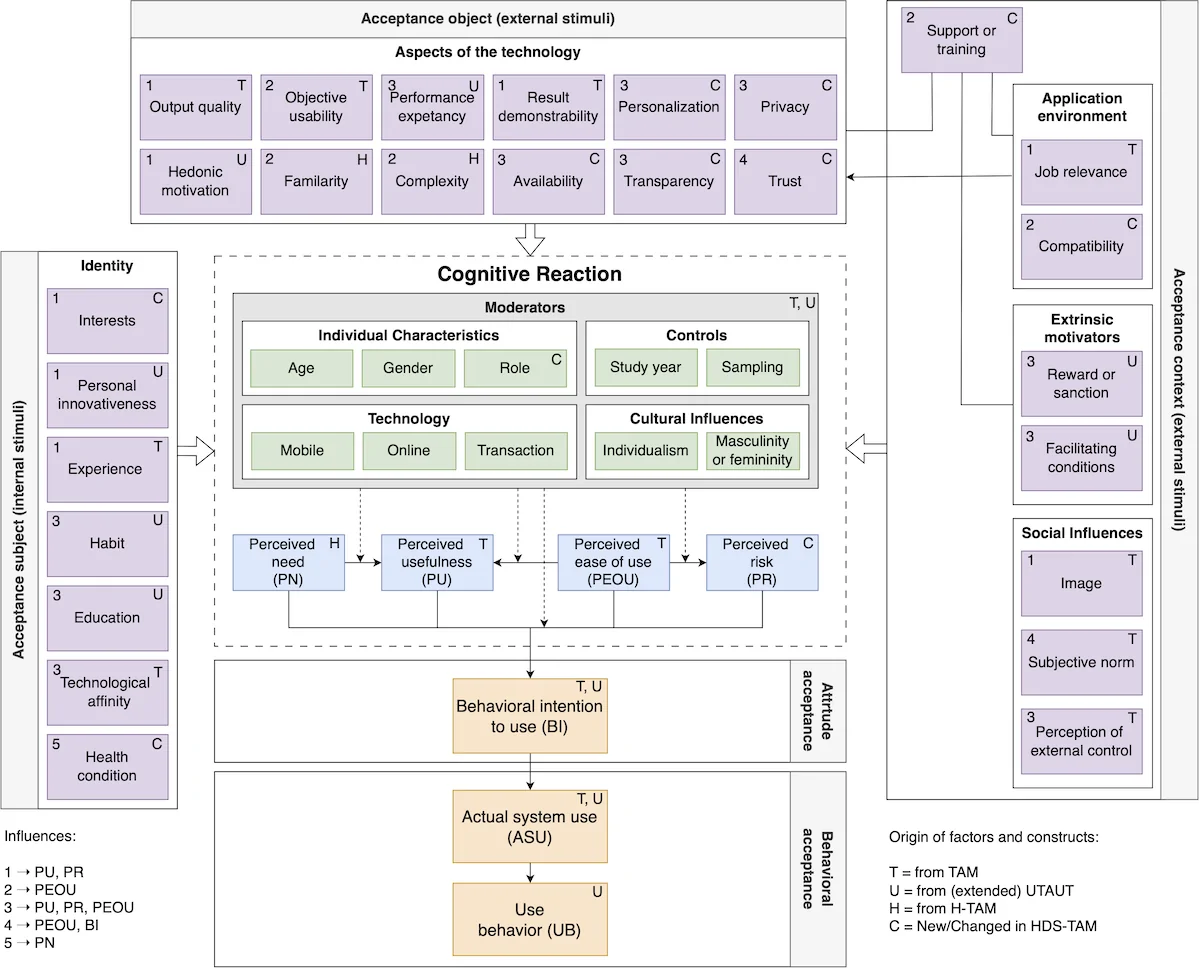
The HDS-TAM. Due to graphical complexity, influences of individual variables are depicted as numbers in the top left corner. The top right corner of the boxes shows the origin of the variables and constructs. H-TAM: health care technology acceptance model; HDS-TAM: health dataspace technology acceptance model; TAM: Technology Acceptance Model; Unified Theory of Acceptance and Use of Technology.

#### Factors of Acceptance and Moderators

HDS-TAM includes PEOU and PU from TAM, perceived need (PN) from H-TAM, and perceived risk (PR) as an additional construct. PN and PR are supported by prior literature (Table 1) [13,30-34]. These factors influence behavioral intention to use (BI). In line with TAM and UTAUT, HDS-TAM further distinguishes between actual system use (ASU), representing initial use, and use behavior (UB), reflecting sustained, occasional, or discontinued use over time.

**Table 1.** Research on the acceptance factors PN^a^ and PR^b^.

Acceptance influencing factor	References
PN	[13,30]
PR	[31-34]

a PN: perceived need.

b PR: perceived risk.

PEOU captures whether technology is easy to understand and use. PU reflects expected benefits, while PR was added to capture expected disadvantages more explicitly. Separating PU and PR allows positive and negative evaluations of a technology to be examined in parallel. As in TAM, PEOU is assumed to influence PU; in HDS-TAM, it is also assumed to affect PR, since complex or difficult systems may increase perceived harm or burden, particularly in medical contexts.

HDS-TAM also distinguishes PN from PU. Following Harris and Rogers [13], PN refers to the belief that technological support is needed. This distinction is especially relevant in health care, where acceptance may depend not only on expected benefits but also on whether the technology is perceived as necessary. Prior studies suggest that illness experience and health status can increase this sense of need, whereas a lack of perceived necessity may reduce acceptance even when benefits are recognized [26,35,36].

As in TAM and UTAUT, HDS-TAM includes moderators that influence the strength of relationships between variables. These were adopted from prior models, except that the consumer/employee distinction was generalized to the moderator role. This allows the model to account for the different perspectives of stakeholders in HDSs, such as patients, physicians, or pharmaceutical companies.

#### Justification

HDS-TAM includes additional factors whose relevance is supported by prior research on technology acceptance. Table 2 provides a brief overview of those variables together with references to papers and studies that confirm their influence on acceptance.

**Table 2.** Overview of related research on new variables in HDS-TAM^a^ in addition to UTAUT^b^ and TAM^c^.

Perspective and variable	References
Identity	
Health condition	[26,37,38]
Personal interests	[39]
Personal innovativeness	[31,40]
Aspects of the technology	
Personalization (patient-centeredness)	[41,42]
Availability	[43,44]
Privacy	[32,33,45]
Trust	[13,18,25,38,45,46]
Application environment	
Compatibility	[13,47]
Technical infrastructure (part of compatibility)	[46,48]
Task-technology fit (part of compatibility)	[33]
Legal aspects (part of compatibility)	[49,50]
Extrinsic motivators	
Reward or sanction	[51,52]
Uncategorized	
Support or training	[33,53,54]

a HDS-TAM: health dataspace technology acceptance model.

b UTAUT: Unified Theory of Acceptance and Use of Technology.

c TAM: Technology Acceptance Model.

The compatibility variable in HDS-TAM has several meanings that also differ from the compatibility concept in UTAUT. Compatibility aspects such as standards, workflows/habits, technological infrastructure, task-technology fit, and legal aspects should be considered. For example, the planned European Electronic Health Record Exchange Format of the EHDS will be a standard that addresses the compatibility of data across Europe and is therefore an important step toward increasing acceptance [55].

When evaluating or predicting the acceptance of a technology, one must be aware of these breakdowns to capture the variables as holistically as possible. For reasons of complexity, further breakdowns were not integrated into the HDS-TAM. However, in concrete studies based on HDS-TAM, these breakdowns should be considered.

#### Concepts of Identity and Technology Acceptance

Research on the acceptance of new technologies has highlighted the impact of self-identity [56]. Identity comprises a multitude of variables, each contributing uniquely to an individual’s composition. Consequently, the weighting of acceptance factors becomes highly individualized.

While factors related to identity typically lead to technology being perceived as enriching, health holds a special significance. The absence of good health instills a strong desire to survive, motivating individuals to embrace new technologies for health improvement [26]. When users face pressing needs that a technology can address, they are more inclined to accept and integrate it into their lives [13]. Particularly in cases of life-threatening illnesses, individuals are almost compelled to adopt technological solutions.

As a result, technologies catering to health deficiencies hold considerable potential for acceptance. Conversely, for individuals in good health, this motivator is absent. They lack the conscious drive to enhance their health, diminishing the perceived benefits of such technologies or rendering them insignificant. Therefore, the persuasive power of this primary domain factor depends on identity and cannot be considered the sole determinant of acceptance for all users. To enhance the acceptance of HDSs, it is imperative to include factors from other perspectives.

### Step 2: HDS-TAM Case Study

#### Development of Scenarios

We used a scenario-based approach with real-world use cases to illustrate specific ePA usage situations. As the study focused on patients, scenarios reflected their perspective. This method captures attitudes and perceptions of concrete usage situations rather than abstract opinions. The advantages of the ePA described by the German Federal Ministry of Health [57] formed the basis for the 8 survey scenarios listed in Table 3 together with the associated questionnaire items.

**Table 3.** Overview of scenario contexts and related questionnaire items.

Scenario	Context	Questionnaire items
1	Overview of diagnoses, findings, and document storage	PN1^a^, PU1^b^, PU2, PU3, PU4, PU5
2	Medication overview due to “eRezept”	PU6, PU7, PR1^c^, BI1^d^, PR2
3	Treatments in emergencies	PU8, PU9, BI2
4	Data protection and individual control	PR3, PR4, PR5, S1^e^
5	Security of health data	PR6, PR7, PR8, T1^f^, T2
6	Use of the ePA^g^ in telemedicine	PR9, BI3, PR10
7	Social influences and perceived need	PN2, PU10, SI1, PU11
8	Secondary use of ePA data for research	BI4, PR11, BI5, PR12, T3, T4, T5
-	Additional questions on experiences and attitudes	PEOU1^h^, PEOU2

a PN: perceived need.

b PU: perceived usefulness.

c PR: perceived risk.

d BI: behavioral intention to use.

e S: support.

f T: transparency.

g ePA: German electronic patient record.

h PEOU: perceived ease of use.

#### Development of Hypotheses

Hypotheses were derived by selecting variables and constructs of the HDS-TAM with a focus on patients using the ePA. The health condition variable was further detailed by the 2 variables for existing health problems and regular medication. Figure 3 illustrates the constructs with the hypotheses and their relationships. The list of the final 20 hypotheses can be found in Multimedia Appendix 1. A total of 18 hypotheses address the influence of variables and constructs on BI and ASU, while two hypotheses examine moderator effects. In the empirical case evaluation, ASU refers specifically to binary self-reported current ePA use. Longer-term UB, as conceptualized in HDS-TAM, was not assessed, as ePA applications were unavailable to most participants at the time of the study.

The questionnaire items listed in Multimedia Appendix 2 address variables and constructs of the hypothesis model. The participants had to rate the statements on a 4-level bipolar Likert scale. The answer options were strongly disagree, disagree, agree, and strongly agree. The scale uses an even number of levels to force a judgment toward one pole or the other. The neutral option is known to capture multiple reasons for selection, which is undesirable [58]. Thus, our study participants had no option to select a neutral response and were required to indicate a tendency. To reduce a possible forced-choice bias, we decided to allow respondents to skip any question.

**Figure 3.**
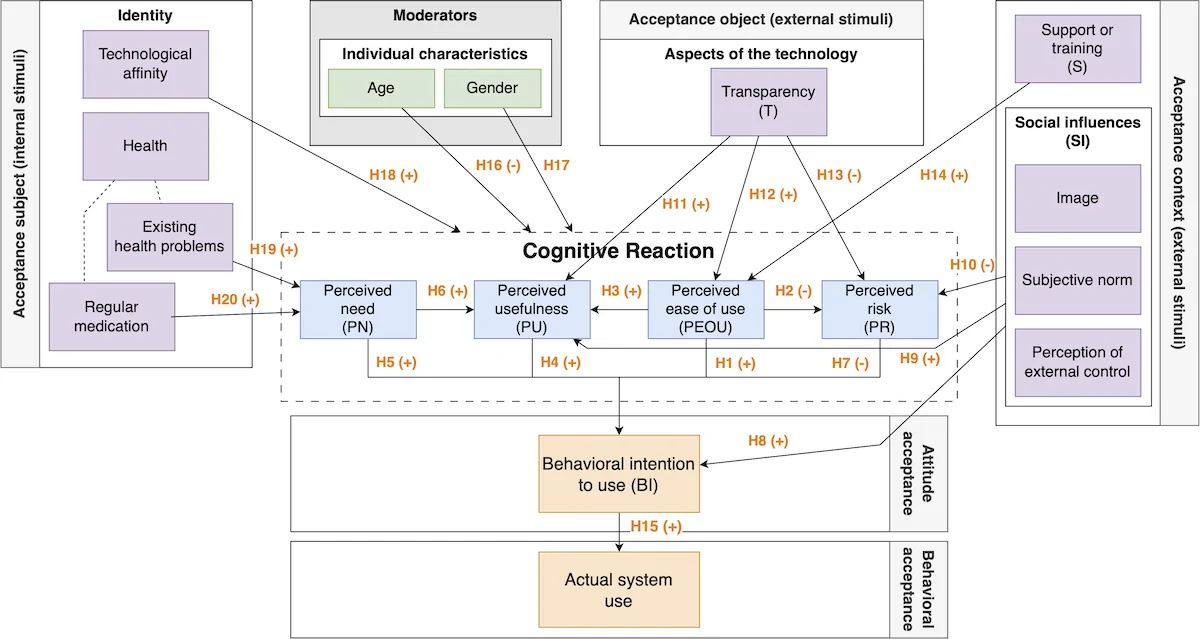
Constructs selected from HDS-TAM for the ePA case evaluation. The arrows illustrate the hypotheses H1-H20 with the direction of the influence. (+) means a positive and (−) a negative correlation. BI: behavioral intention to use; ePA: German electronic patient record; H: hypothesis; HDS-TAM: health dataspace technology acceptance model.

#### Development of Questionnaire

The questionnaire collected sociodemographic information about age, gender, occupation, actual ePA usage, previous illness, regular medication, and technical knowledge. The 8 scenarios were then presented to participants, each with a brief introduction and the related questionnaire items shown in Table 3. The questionnaire concluded with 2 items, PEOU1 and PEOU2, capturing PEOU, as these could not be linked to a scenario. Finally, participants answered 2 open questions on advantages motivating ePA use and on concerns or obstacles. To exclude random or suspicious answers, the questionnaire contains an attention check item that asks respondents to check the first answer strongly disagree. Because the survey addressed the ePA from the perspective of potential users in Germany, the questionnaire was administered in German. The complete questionnaire, together with an English translation, can be found in Multimedia Appendix 3.

#### Online Survey Procedure

Using the SoSci Survey, we created a shareable survey link. The survey was conducted by the authors, supported by a graduate student research internship. Initially, all University of Koblenz students were invited via a mailing list and encouraged to share the link with others, including acquaintances. The link was also distributed through social media channels such as Instagram and WhatsApp. Test runs showed that completing the survey took around 15 minutes.

The survey took place from March 9 to  April 7, 2025. Participants were informed about the study’s aim, duration, anonymity, and voluntary nature. Before starting, consent was collected. Respondents could end participation and skip items at any time without explanation. Respondents received no incentives. In total, the survey was accessed 235 times, and 163 questionnaires were submitted. Of these, 149 met the inclusion criteria and passed the attention-check item. Item-level nonresponse was permitted and is analyzed below.

#### Data Analysis

In the final phase of the case study, the survey data were exported from SoSci Survey to IBM SPSS Statistics (version 30) and R (version 4.6.1; R Foundation) with MICE (version 3.19.0; Multiple Imputation by Chained Equations) and cSEM (version 0.6.1) for analysis and interpretation. Likert-scale responses were coded numerically from 1 (strongly disagree) to 4 (strongly agree).

Data analysis proceeded in several stages. First, we conducted an analysis of the nonresponses. Items were recoded where necessary to ensure consistent interpretation of scale directions. Binary yes/no items were recoded to 1 and 0. Descriptive statistics, including means, SDs, and response distributions, were computed for all study variables. Interitem correlation matrices and Cronbach α coefficients were examined to assess preliminary evidence of internal consistency reliability. In addition, exploratory factor analysis (EFA) was conducted to evaluate the dimensionality of the measurement instruments and to identify potential departures from the theoretically expected factor structure. Factor loadings of at least 0.4 were considered acceptable, and values above 0.7 were considered very good. Construct reliability was assessed using Cronbach α, with 0.7 as the threshold [59]. Before factor analysis, data suitability was assessed using the Kaiser-Meyer-Olkin (KMO) criterion and Bartlett test of sphericity [60]. For samples between 100 and 200, KMO values of 0.5‐0.6 are considered acceptable, and values above 0.8 indicate very good suitability. Bartlett test significance values below 0.05 were regarded as sufficient. These preliminary analyses were performed using the original observed data.

Prior to structural equation modeling, based on the nonresponse analysis, missing values were addressed using MICE. Following data imputation, partial least squares structural equation modeling (PLS-SEM) was used to estimate and assess the measurement and structural models. The results for all imputations were calculated and subsequently pooled according to Rubin rules (RR). The measurement model was evaluated through the inspection of indicator loadings, composite reliability (ρC), and average variance extracted (AVE), providing evidence of indicator reliability, internal consistency reliability, and convergent validity. Discriminant validity was assessed using the heterotrait-monotrait ratio of correlations (HTMT). For the composite constructs PN and T, indicator collinearity was assessed using variance inflation factors (VIFs). Because internal-consistency measures such as Cronbach α and ρC are not appropriate criteria for evaluating composite specifications, these indices were not used to assess PN and T in the final PLS-SEM model.

To assess whether the available sample provided adequate sensitivity for the structural model, we conducted a regression-based sensitivity analysis using the most complex endogenous equation in the core model, behavioral intention, which included 4 predictors. With 149 answers, *α*=.05, and 80% statistical power, the minimum detectable effect size was approximately *f*²=0.08. Thus, the available sample provided reasonable sensitivity for medium effects but limited sensitivity for small effects. This analysis was used as an approximate power benchmark for the structural model rather than as a formal validation of PLS-SEM sample adequacy.

Following multiple imputation, the PLS-SEM model was estimated separately in each of the 20 imputed datasets. For structural path coefficients and other model parameters for which an estimate and SE were available, results were combined using RR, accounting for both within-imputation and between-imputation variability. Measurement and model-evaluation indices for which conventional Rubin pooling is not directly applicable, including AVE, ρC, HTMT, VIF, *R*², and *f*², were calculated separately for each imputed dataset and summarized across imputations using their arithmetic means. Variability of these indices across imputations was additionally inspected to assess the stability of the results. After satisfactory measurement properties had been established, the structural model was evaluated by estimating the hypothesized relationships among latent constructs (H1-H14). An exploratory PLS-SEM analysis was conducted to investigate the potential influence of technological affinity (H18) as well as the influence of health condition (H19) and regular medication (H20) on PN. H18 concerned the hypothesized effect of technological affinity on PEOU. Additional paths from technological affinity to PU and PR were examined exploratorily and were not considered tests of H18. To examine the relationship between BI and self-reported actual ePA use (H15), a binary logistic regression analysis was performed. Finally, potential moderating effects (H16 and H17) were investigated in an exploratory analysis using linear regression with latent construct scores and interaction terms.

The questionnaire also included 2 open-ended questions on perceived benefits, concerns, and barriers regarding ePA use. These responses were analyzed qualitatively by extracting and grouping the main statements into thematic categories.

## Results

### Overview

The literature review and model-building process yielded HDS-TAM as the conceptual basis for the case evaluation. The following sections report the empirical results of its patient-focused application to the ePA.

### Sample Description

The questionnaire was accessed 235 times, and 163 participants began answering questions on the ePA and submitted responses. After excluding 14 responses that failed the attention-test item and showed additional anomalies, 149 valid datasets remained for analysis; 111 datasets were complete. Multimedia Appendix 4 provides detailed sample characteristics.

The sample was heterogeneous but unevenly distributed. Participants younger than 30 years accounted for 67.8% (n=101) of the sample, largely because many respondents were students. Women were overrepresented (59.7%, n=89) compared with men (33.6%, n=50); 1 respondent reported a diverse gender, while 9 participants did not report gender. A total of 53.7% (n=80) of the sample were university students. At the time of the survey, 23.5% (n=35) of participants reported actual use of the ePA, and 113 did not actually use the ePA. One answer was missing. Further, 27.5% (n=41) reported previous illnesses, and 33.6% (n=50) used regular medication. Technical knowledge was also unevenly distributed, with most respondents (94%, n=140) reporting medium to high levels and only 9 indicating low or very low technical knowledge.

### Responses

Figure 4 shows the distribution of survey responses. In the following, the responses are summarized regarding the factors influencing the acceptance of the ePA.

- Regarding behavioral intention (BI1-5), participants tended to give affirmative answers toward BI, which indicates a generally positive attitude toward its use.
- Regarding PEOU1-2, most participants agreed that using the ePA (n=98) and understanding its functionality (n=101) were easy.
- Regarding PN1-2, responses to both PN items were predominantly positive.
- Regarding PR1-12, responses to PR items were heterogeneous. Data security is very important to participants, while there is a generally high level of trust in the security of the ePA. However, several negative responses suggested that PR remained high for a subset of respondents.
- Regarding PU1-11, PU responses were heterogeneous as well. The emergency-use scenario stood out particularly, with only 5 participants disagreeing or strongly disagreeing. Overall, PU was rated very positively.
- Regarding transparency (T1-5), responses were mixed. Responses regarding the information provided on data processing and associated risks tended to be negative, suggesting that transparency is lacking in this area. Responses on data use indicate that this is an important concern. Although confidence in data sharing for research purposes was generally positive, it is nevertheless divided: 44 participants answered this negatively.
- Regarding support (S1), 89 participants indicated that they would appreciate support when managing access permissions and configuring the ePA.
- Regarding SI1, 88 participants tended to disagree that other individuals influenced their decision to use the ePA.

Overall, the answers are rather positive, although, except for PU, there were always some negative answers to the questions.

To ensure consistent scale directions, the items PR1, PR4, PR6, PR8, PR9, and T1 had to be inverted. This is indicated by appending an “i” to the item name, for example, PR1i.

**Figure 4.**
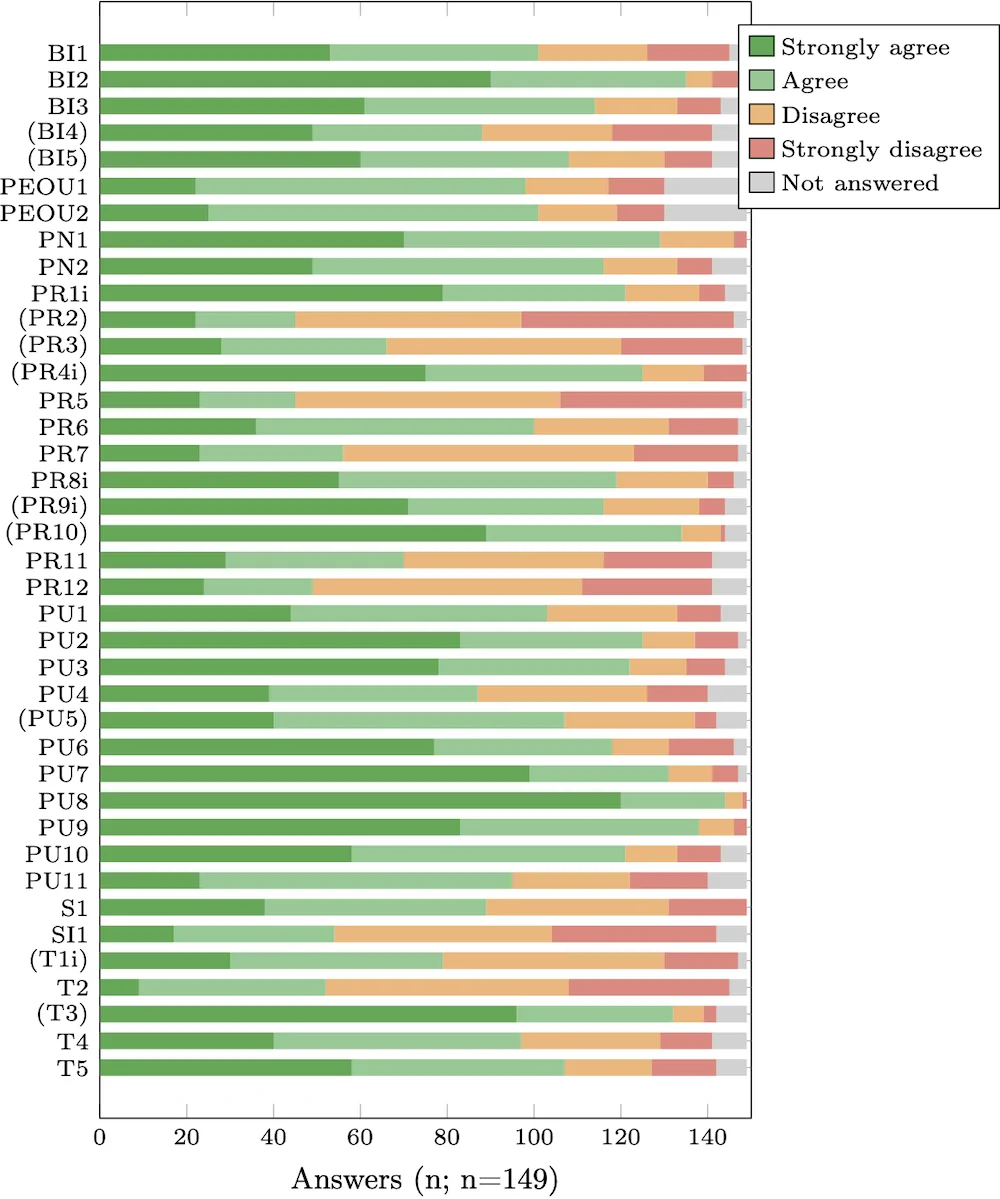
Response counts for the individual items. Items in brackets were excluded from the PLS-SEM model. Rationales for exclusion are provided in the text. BI: behavioral intention to use; PEOU: perceived ease of use; PLS-SEM: partial least squares structural equation modeling; PN: perceived need; PR: perceived risk; PU: perceived usefulness; S: support; SI: social influence; T: transparency.

### Nonresponse Analysis

Nonresponses were limited overall, with 216 of 6705 (3.22%) item-level observations missing. Most indicators exhibited less than 6% missingness. The highest proportion was observed for the PEOU items (19 nonresponses, 12.75% each). Examination of the missing-data pattern indicated that omission of the PEOU items was significantly associated with ePA use status (Fisher exact test: *P*=.046 for both PEOU1 and PEOU2), with nonusers showing a missingness count of 18 while ePA users only contributed 1 missing value. In contrast, missingness in other indicators was not systematically related to ePA use status. Four respondents skipped questionnaire pages, thereby contributing 32.9% (n=71) of the missing values. Consequently, the assumption of MCAR (missing completely at random) was not supported. Because the observed missingness pattern could largely be explained by measured variables included in the dataset, a MAR (missing at random) mechanism was considered plausible. However, as with any missing-data analysis based on observed data, an MNAR (missing not at random) mechanism cannot be ruled out conclusively. Missing values were therefore handled using MICE, including all study variables in the imputation model. Multiple imputation was preferred over listwise deletion because the latter would have systematically reduced representation of nonusers of the ePA and thereby increased the risk of biased parameter estimates. Listwise deletion would have excluded 38 respondents, reducing the analytic sample to 111. Missing data details are provided in Multimedia Appendix 5.

### Descriptive Analysis

Preliminary descriptive analyses indicated satisfactory item characteristics. All indicators used the full 4-point response scale and exhibited adequate variability (SD 0.52‐1.09). Item means suggested generally favorable perceptions of usefulness and behavioral intention. Distributional properties were largely acceptable, with most skewness and kurtosis values remaining within conventional thresholds. One item (PU8) displayed a pronounced ceiling effect (mean 3.77, SD 0.52, skewness=−2.43, kurtosis=6.36), indicating a strong concentration of responses at the highest scale point. Given the robustness of PLS-SEM to nonnormal data and the absence of severe distributional abnormalities across the remaining indicators, all items were retained for subsequent psychometric evaluation.

### Interitem Correlation

Inspection of the interitem correlation matrix for PU revealed predominantly moderate to strong positive associations among indicators (*r*=0.33 to 0.78), supporting the assumption that the items capture a common latent construct. No correlations exceeded .80, indicating an absence of problematic redundancy. However, item PU5 exhibited consistently weak correlations with the remaining indicators (*r*=0.02 to 0.25), suggesting limited shared variance with the construct.

PR items revealed a substantial cluster of moderate-to-strong positive associations among most indicators (*r*=0.25 to 0.74), providing preliminary evidence of construct coherence. Particularly strong relationships were observed among PR5, PR6i, PR7, PR11, and PR12. In contrast, item PR4i displayed consistently weak or negative correlations with several indicators (*r*=−0.19 to 0.30), suggesting limited shared variance with the remaining items. PR10 and, to a lesser extent, PR1i and PR9i, also exhibited comparatively weaker relationships.

Examination of the interitem correlations revealed limited coherence among the transparency indicators. While T3 and T5 showed a substantial positive association (*r*=0.53), several item pairs exhibited weak or negative correlations. Inspection of the item statements suggests that the indicators may capture distinct concepts, including transparency of information (T2), PR awareness (T1), trust in data handling (T4), and user control over research use of data (T3 and T5).

The BI indicators exhibited moderate to strong positive interitem correlations (*r*=0.34 to 0.77), providing preliminary evidence of internal consistency. All item pairs were positively related, and no correlation exceeded commonly accepted thresholds for item redundancy. The strongest association was observed between BI4 and BI5 (*r*=0.77), whereas the weakest relationship occurred between BI2 and BI4 (*r*=0.34).

The 2 PN indicators exhibited a weak positive correlation (*r*=0.25). Although the association was in the expected direction, its magnitude was notably lower than that observed for most other constructs.

### Reliability Analysis

Cronbach α values are reported in Table 4. PU, PR, PEOU, and BI showed acceptable reliability. In contrast, PN (*α*=0.40) and transparency (*α*=0.22) did not reach the recommended threshold of 0.70.

**Table 4.** Reliability test of the constructs by Cronbach α.

Construct	Cronbach α	95% CI
PU^a^	0.90	0.88 to 0.93
PR^b^	0.85	0.82 to 0.89
PEOU^c^	0.85	0.79 to 0.89
PN^d^	0.40	0.18 to 0.57
T^e^	0.22	0.00 to 0.40
BI^f^	0.83	0.79 to 0.87

a PU: perceived usefulness.

b PR: perceived risk.

c PEOU: perceived ease of use.

d PN: perceived need.

e T: transparency.

f BI: behavioral intention to use.

### EFA

Data from all constructs were included in the EFA. The KMO measure of sampling adequacy indicated excellent suitability for factor analysis (KMO=0.91). The Bartlett test of sphericity was significant (*χ*²_666_=3740.07, *P*<.001), indicating that the correlation matrix differed significantly from an identity matrix and was therefore suitable for factor analysis.

Parallel analysis suggested a 5-factor solution. The solution broadly supported the existence of factors corresponding to PU or acceptance, PR, BI, PEOU, and a control- or governance-related dimension. In contrast, the T indicators did not form a coherent factor and exhibited weak and unstable loadings across all solutions. Similarly, the PN indicators failed to load consistently on a common factor.

Based on reliability analyses, theoretical assessment, and EFA, items PR2, PR4i, PR9i, and PR10 were removed from the PR construct before subsequent PLS-SEM measurement-model assessment.

For the T items, EFA did not support a unidimensional reflective measurement structure. T1i and T3 related to knowledge and control and were excluded because of this theory-driven argument.

PN and T were initially operationalized as multi-item constructs without an a priori composite specification. After the reflective measurement diagnostics indicated poor internal consistency and the EFA did not support stable unidimensional structures, the retained indicators were reassessed conceptually. PN and T were subsequently specified as composite constructs because their indicators represent distinct but complementary components of the underlying concepts rather than interchangeable manifestations of a common latent variable. T encompasses information provision (T2), trust in research-related data use (T4), and notification mechanisms regarding data access (T5), whereas PN reflects both the desire to prepare for medical consultations through access to records (PN1) and the perceived necessity of digital health data storage (PN2). Because changes in one indicator do not necessarily imply corresponding changes in the others, high interitem correlations are not theoretically required. The respecification was therefore data-informed and theory-guided and should be prospectively evaluated in an independent sample.

Further item refinement was guided by both theoretical considerations and empirical evidence. Removal decisions were based on conceptual inconsistency with the intended construct and evidence of poor psychometric performance. Item refinement was therefore exploratory and based on both psychometric evidence and theoretical assessment rather than on statistical criteria alone. PU5 was removed because it demonstrated insufficient loading on the PU factor during EFA. The item’s inclusion reduced construct homogeneity and convergent validity. PR3 was excluded because it measured users’ perceived ability to manage access rights, which is conceptually closer to competence and control beliefs than to PR. Likewise, BI4 and BI5 were removed because they addressed willingness to share health data for research purposes rather than intention to use the ePA. Removal of these indicators improved construct interpretability while preserving theoretical content validity. The final list of indicators is shown in the PLS-SEM model in the following section.

### Data Completion

Before model estimation, missing values were addressed using MICE with predictive mean matching. The imputation model included all study variables and the procedure generated 20 imputed datasets using 5 MICE iterations. To evaluate the influence of the imputation procedure, the final model was later re-estimated using complete-case data. Parameter estimates, reliability measures, convergent validity statistics, and explained variances were highly comparable between the multiply imputed and complete-case datasets. In particular, the substantive conclusions regarding the effects of T, PN, PU, and PR remained unchanged. These findings suggest that the reported results are robust to the treatment of missing data.

### PLS-SEM Estimation

The PLS-SEM core model derived from HDS-TAM was designed as follows. The measurement model defines the following reflective constructs:

 BI: BI1, BI2, BI3

 PEOU: PEOU1, PEOU2

 PU: PU1, PU2, PU3, PU4, PU6, PU7, PU8, PU9, PU10, PU11

 PR: PR1i, PR5, PR6i, PR7, PR8i, PR11, PR12

PN and T were defined as composite constructs:

 PN: PN1, PN2

 T: T2, T4, T5

According to HDS-TAM, the dependencies in the structural model were defined as:

 PEOU: T

 PU: T+PEOU+PN

 PR: T+PEOU

 BI: PU+PR+PEOU+PN

### Measurement Model

The measurement model assessment presented in Table 5 was highly stable across the 20 imputed datasets. For AVE, ρC, HTMT, *R*^2^, and VIF estimates, we pooled using mean values, and the variability across imputations was negligible (SD ≤0.03 for all estimates). All reflective constructs showed satisfactory internal consistency reliability, with ρC ranging from 0.899 to 0.938 and AVE values exceeding the recommended threshold of 0.50. PN and T were specified as composites and were therefore evaluated separately from the reflective constructs. Indicator collinearity was low for both composites (all VIFs ≤1.27), providing no indication of problematic redundancy among the retained composite indicators. Indicator loadings pooled according to RR were statistically significant and generally above 0.70.

The evaluation of discriminant validity yielded mixed evidence. While most values were below the liberal threshold of 0.90, the HTMT value of 0.981 between PU and BI exceeded commonly recommended cutoffs, indicating limited discriminant validity between these constructs.

**Table 5.** Pooled reliability, convergent validity, and discriminant validity. Pooling of 20 imputations with mean values. For HTMT^a^, off-diagonal elements represent HTMT ratios. Recommended thresholds: AVE^b^ >0.50; ρC^c^ >0.70; HTMT <0.85 (strict criterion) or <0.90 (liberal criterion).

Construct	AVE	Composite reliability (ρC)	1	2	3	4
PEOU^d^	0.876	0.929	1.000	—^e^	—	—
PU^f^	0.605	0.938	0.628	1.000	—	—
PR^g^	0.623	0.919	0.573	0.872	1.000	—
BI^h^	0.749	0.899	0.614	0.981	0.866	1.000

a HTMT: heterotrait-monotrait ratio of correlations.

b AVE: average variance extracted.

c ρC: composite reliability (Jöreskog ρ).

d PEOU: perceived ease of use.

e Not available.

f PU: perceived usefulness.

g PR: perceived risk.

h BI: behavioral intention to use.

### Structural Model

Prior to testing the hypotheses, collinearity among predictor constructs was assessed. All structural VIF values were below 4.1, indicating that multicollinearity was not a concern.

The model explained 20.7% of the variance in PEOU, 67% in PU, 54% in PR, and 75.9% in BI.

Table 6 summarizes the direct and indirect effects. T was positively associated with PEOU (*β*=.454, *P*<.001) and PU (*β*=.220, *P*=.001) and negatively associated with PR (*β*=−.594, *P*<.001). PN showed a strong positive association with PU (*β*=.567, *P*<.001), whereas PEOU showed a weaker positive association with PU (*β*=.193, *P*=.004) and a negative association with PR (*β*=−.240, *P*=.002). Regarding BI, PU showed the largest positive association (*β*=.670, *P*<.001), whereas PR showed a smaller negative association (*β*=−.149, *P*=.04). The direct associations of PN and PEOU with BI were not statistically significant.

**Table 6.** Pooled direct and indirect effects. The PLS-SEM^a^ model was estimated separately in each of the 20 imputed datasets using 5000 bootstrap resamples. Path coefficients and their corresponding within-imputation variances were subsequently combined across imputations using Rubin rules. Effect size cutoffs: <0.02 negligible, 0.02 to <0.15 small, 0.15 to <0.35 medium, ≥0.35 large.

Relationship	*β*	SE	*t* ^b^	*P* value	95% CI	*f* ^2^	Result
Direct effects							
T^c^ → PEOU^d^	.454	0.071	6.361	<.001^e^	0.314 to 0.594	0.262	Supported
T → PU^f^	.220	0.068	3.262	.001^e^	0.088 to 0.353	0.106	Supported
PN^g^ → PU	.567	0.055	10.321	<.001^e^	0.460 to 0.675	0.674	Supported
PEOU → PU	.193	0.067	2.871	.004^e^	0.061 to 0.325	0.080	Supported
T → PR^h^	−.594	0.069	−8.638	<.001^e^	−0.728 to −0.459	0.609	Supported
PEOU → PR	−.240	0.077	−3.108	.002^e^	−0.391 to −0.089	0.101	Supported
PN → BI^i^	.071	0.076	0.927	.35^e^	−0.079 to 0.220	0.009	Not supported
PEOU → BI	.038	0.060	0.630	.53^e^	−0.079 to 0.154	0.005	Not supported
PU → BI	.670	0.091	7.353	<.001^e^	0.491 to 0.848	0.457	Supported
PR → BI	−.149	0.072	−2.085	.04^e^	−0.290 to −0.009	0.034	Supported
Indirect effects							
T → PU	.088	0.037	2.346	.02^e^	0.014 to 0.161	—^j^	Supported
T → PR	−.109	0.042	−2.588	.01^e^	−0.192 to −0.026	—^j^	Supported
T → BI	.329	0.058	5.694	<.001^e^	0.216 to 0.442	—^j^	Supported
PN → BI	.380	0.058	6.587	<.001^e^	0.267 to 0.493	—^j^	Supported
PEOU → BI	.165	0.056	2.971	.003^e^	0.056 to 0.274	—^j^	Supported

a PLS-SEM: partial least squares structural equation modeling.

b Reported *t *values are cSEM bootstrap results that do not have associated degrees of freedom (*df*) as in standard *t* tests.

c T: transparency.

d PEOU: perceived ease of use.

e Significant P values.

f PU: perceived usefulness.

g PN: perceived need.

h PR: perceived risk.

i BI: behavioral intention to use.

j Effect size for indirect paths is not available.

The effect size analysis for the direct paths indicates that only a subset of the statistically significant relationships was substantively important. The effect-size analysis of the direct structural paths showed substantial differences in the magnitude of the observed associations. The PU→BI association had a large *f*² value (*f*²=0.457), as did PN→PU (*f*²=0.674) and T→PR (*f*²=0.609). The remaining statistically significant direct paths showed small to moderate *f*² values (*f*²=0.034-0.262). The largest total indirect associations with BI were observed for PN (*β*=.380, *P*<.001) and T (*β*=.329, *P*<.001), whereas the total indirect association of PEOU with BI was smaller (*β*=.165, *P*=.003). Because bootstrap estimates were obtained only for total indirect effects, individual mediation pathways were not assessed separately. Overall, the estimated indirect associations indicate that several relationships of PN, T, and PEOU with BI involved pathways through PU. These findings should be interpreted cautiously given the limited discriminant validity between PU and BI. As a robustness check, the PLS-SEM model was re-estimated using the complete-case sample. The results closely mirrored those obtained from the multiply imputed dataset. Reliability and convergent validity remained satisfactory; the explained variance of the endogenous constructs changed only marginally (*R*² differences <0.02), and the estimated total effects were highly similar across both analyses. These findings suggest that the study’s conclusions are robust to the treatment of missing data and are not materially affected by the use of imputation.

### Additional Indicators From HDS-TAM

As an exploratory analysis, the structural model was extended by incorporating the indicators regular medication use, health issues, support, SI, and technological affinity from HDS-TAM as additional antecedents:

PN: Medication+HealthIssues

PEOU: *t*+Support+TechAffinity

PU: *t*+PEOU+PN+SI+TechAffinity

PR: *t*+PEOU+SI+TechAffinity

BI: PU+PR+PEOU+PN+SI

The extended model produced only minor changes in explained variance (*R*²PU=0.679 vs 0.670; *R*²PR=0.548 vs 0.540; *R*²BI=0.750 vs 0.759) and did not meaningfully improve the explanatory performance of the core model. The additional variables, technological affinity, medication use, health issues, support, and SI showed no significant direct effects on any of the focal constructs or on BI. These findings suggest that in our survey the explanatory power of the model is primarily driven by the theoretically derived UTAUT/TAM-related constructs, with limited incremental contribution from the additional exploratory variables.

### Association Between BI and Self-Reported Actual ePA Use

To examine the association between BI and self-reported actual ePA use, a logistic regression was estimated using the latent BI score as the predictor and self-reported ePA use as the dependent variable. Although the estimated coefficient was positive (*β*=.317, *P*=.15, 95% CI −0.113 to 0.748), the coefficient was not statistically significant. A 1-unit increase in BI was associated with approximately 37% higher odds of ePA use (OR=1.37, *P*=.15, 95% CI 0.89 to 2.11). Consistent with this result, users of the ePA reported somewhat higher BI (mean 3.33, SD 0.61) than nonusers (mean 3.11, SD 0.85), but the difference did not reach conventional levels of statistical significance (*t*_77.90_=−1.69, *P*=.10). Thus, the estimated association between BI and self-reported actual ePA use was positive but imprecise and not statistically significant in the present sample. Given the small number of actual users, this analysis should be considered preliminary.

### Moderation by Age and Gende*r*

Potential moderating effects of age and gender were explored through interaction models. The sample was divided into younger (≤29 y) and older respondents (≥30 y), as well as female and male participants.

To formally probe moderation, interaction terms between the strongest predictor, PU, and gender and between PU and age group were estimated using bootstrapped construct scores. The results were pooled across the 20 imputations according to RR. Neither the interaction with gender (*β*=0.028, *P*=.75, 95% CI −0.142 to 0.198) nor the interaction with age (*β*=−0.056, *P*=.55, 95% CI −0.237 to 0.126) were statistically significant. Likewise, the corresponding main effects of gender (*P*=.44) and age group (*P*=.88) were not significant.

Overall, the findings for the present sample provide no evidence that the relationship between PU and BI differed systematically across gender or age groups.

Table 7 summarizes the statistical findings with respect to the original hypotheses. In total, 8 out of 20 hypotheses are supported by the results. In addition, 5 statistically significant total indirect effects were observed.

**Table 7.** Summary of findings for original hypotheses H1^a^-H20. Direction is positive (+) or negative (−). Results are discussed in detail in the next section. Hypotheses in braces were not part of the core structural model and were included in the exploratory analysis.

Hypothesis	Influence or direction	*β*	*P* value	95% CI	*f* ^2^	Supported
H1	PEOU^b^→BI^c^ +	.038	.53	−0.079 to 0.154	0.005	No
H1	PEOU→BI + (indirect)	.165	.003^d^	0.056 to 0.274	—^e^	—^f^
H2	PEOU→PR^g^ −	−.240	.002^d^	−0.391 to −0.089	0.101	Yes^d^
H3	PEOU→PU^h^ +	.193	.004^d^	0.061 to 0.325	0.080	Yes^d^
H4	PU→BI +	.670	<.001^d^	0.491 to 0.848	0.457	Yes^d^
H5	PN^i^→BI +	.071	.35	−0.079 to 0.220	0.009	No
H5	PN→BI + (indirect)	.380	<.001^d^	0.267 to 0.493	—^e^	—^f^
H6	PN→PU +	.567	<.001^d^	0.460 to 0.675	0.674	Yes^d^
H7	PR→BI −	−.149	.04^d^	−0.290 to −0.009	0.034	Yes^d^
(H8)	SI^j^→BI +	.051	.38	−0.065 to 0.164	—^e^	No
(H9)	SI→PU +	.030	.54	−0.067 to 0.125	—^e^	No
(H10)	SI→PR −	−.042	.51	−0.164 to 0.081	—^e^	No
H11	T^k^→PU +	.220	.001^d^	0.088 to 0.353	0.106	Yes^d^
H12	T→PEOU +	.454	<.001^d^	0.314 to 0.594	0.262	Yes^d^
H13	T→PR −	−.594	<.001^d^	−0.728 to ‐0.459	0.609	Yes^d^
H13	T→BI + (indirect)	.329	<.001^d^	0.216 to 0.442	—^e^	—^f^
H13	T→PR − (indirect)	−.109	.01^d^	−0.192 to ‐0.026	—^e^	—^f^
H13	T→PU + (indirect)	.088	.02^d^	0.014 to 0.161	—^e^	—^f^
(H14)	Support→PEOU +	−.012	.88	−0.165 to 0.139	—^e^	No
H15	BI→ASU^l^ +	.317	.15	−0.113 to 0.748	—^e^	No
H16	Age moderates PU→BI −	−.056	.55	−0.237 to 0.126	—^e^	No
H17	Gender moderates PU→BI	.028	.75	−0.142 to 0.198	—^e^	No
(H18)	Technological affinity→PU +	.094	.08	−0.014 to 0.198	—^e^	No
(H18)	Technological affinity→PR +	−.064	.29	−0.185 to 0.055	—^e^	—^f^
(H18)	Technological affinity→PEOU +	−.092	.16	−0.221 to 0.037	—^e^	—^f^
(H19)	Health issues→PN +	−.089	.34	−0.267 to 0.088	—^e^	No
(H20)	Regular medication→PN +	.060	.49	−0.115 to 0.231	—^e^	No

a H: hypothesis.

b PEOU: perceived ease of use.

c BI: behavioral intention to use.

d Significant *P* values.

e Effect size for indirect and nonsignificant effects is not available.

f Hypothesis support is only reported for direct effects in the core model.

g PR: perceived risk.

h PU: perceived usefulness.

i PN: perceived need.

j SI: social influence.

k T: transparency.

l ASU: actual system use.

### Evaluation of Open Questions

The responses to the open-ended questions largely support the statistical findings. All answers are provided in Multimedia Appendix 6.

The most reported advantage of the ePA was the centralized storage of personal health data, which respondents associated with reduced organizational effort. Some also highlighted its usefulness in emergencies, improved communication between doctors, and easier digital access to information.

Reported obstacles were more diverse. The most frequent concerns related to data security, especially data leaks, cyberattacks, and storage on central servers. Some respondents therefore preferred local storage. Others feared data misuse, such as the sale of data or access by insurance companies that could disadvantage high-risk patients. Technical failure and data loss were also mentioned, though these were generally seen as less critical than privacy and leakage risks. In addition, some respondents were concerned that doctors and practices might not be able to use the ePA properly, or that increasing reliance on the system could create problems if it became unavailable.

## Discussion

### Principal Findings

This study addressed a gap in current acceptance research by developing the HDS-TAM (RQ1) and applying it in a case evaluation of the ePA (RQ2). As a case evaluation, HDS-TAM was adapted to the patient perspective and applied to the ePA, which represents a relevant HDS technology because it combines direct patient-facing functionality with broader issues of data access, control, and reuse.

The results provide only partial support for the HDS-TAM framework, with 8 of 20 hypotheses supported.

The very high HTMT of 0.981 between PU and BI gives rise to measurement limitations and raises a discriminant-validity concern. Although a strong association between PU and BI is theoretically expected, this does not resolve the observed lack of discriminant validity [8,61,62]. Respondents generally expressed positive attitudes toward using the ePA. Low discrimination between PU and BI may be a consequence of only little actual use of the system, and respondents may have answered based on assumptions rather than actual experience. This could further increase the correlation between PU and BI in our sample.

Among the modeled predictors, PU showed the largest estimated association with BI (H4, *β*=.670, *P*<.001, 95% CI 0.491 to 0.848, *f*²=0.457), whereas PR showed a small negative association (H7, *β*=−.149, *P*=.04, 95% CI −0.290 to −0.009, *f*²=0.034). However, the magnitude of the PU→BI coefficient should be interpreted in light of the limited discriminant validity between PU and BI. The model explained substantial proportions of variance in BI (*R*²=0.759), PU (*R*²=0.670), and PR (*R*²=0.540). T showed positive associations with PU (H11, *β*=.220, *P*=.001, 95% CI 0.088 to 0.353, *f*²=0.106) and PEOU (H12, *β*=.454, *P*<.001, 95% CI 0.314 to 0.594, *f*²=0.262) and a strong negative association with PR (H13, *β*=−.594, *P*<.001, 95% CI −0.728 to −0.459, *f*²=0.609). Likewise, PN showed a strong positive association with PU (H6, *β*=.567, *P*<.001, 95% CI 0.460 to 0.675, *f*²=0.674). PEOU showed smaller associations with PR (H2, *β*=−.240, *P*=.002, 95% CI −0.391 to −0.089, *f*²=0.101) and PU (H3, *β*=.193, *P*=.004, 95% CI 0.061 to 0.325, *f*²=0.080). Several theoretically expected direct relationships were not supported, including PEOU→BI (H1, *β*=.038, *P*=.53, 95% CI −0.079 to 0.154, *f*²=0.005) and PN→BI (H5, *β*=.071, *P*=.35, 95% CI −0.079 to 0.220, *f*²=0.009). Significant total indirect associations with BI were observed for PEOU, PN, and T.

Our results are largely consistent with prior research on technology acceptance in health care settings [39,45,63]. The role of PU as a key predictor of BI can also be found in related work [10,24,39,45,63,64]. Prior research has likewise emphasized privacy concerns, PR, and trust as important determinants of health care technology acceptance [31-34,45,61,62]. This supports the decision to include the PR construct explicitly in HDS-TAM.

Two deviations from established technology acceptance models deserve particular attention. First, PEOU did not exert a significant direct effect on BI (H1). This differs from the direct relationship hypothesized in the present HDS-TAM case model and from health care–oriented TAM adaptations in which ease of use is considered an antecedent of intention [8,10,13]. Importantly, however, the absence of a direct effect does not imply that ease of use was irrelevant. PEOU was positively associated with PU (*β*=.193, *P*=.004) and negatively associated with PR (*β*=−.240, *P*=.002). In addition, PEOU showed a significant total indirect association with BI (*β*=.165, *P*=.003). Thus, the observed pattern was predominantly indirect with respect to BI, involving pathways through PU and PR. This pattern is compatible with the central role attributed to usefulness in TAM and with prior health care acceptance research showing that the relative importance and pathways of individual determinants vary across technologies and application contexts [10,24].

Second, SI did not significantly predict BI (H8, *β*=.051, *P*=.38, 95% CI −0.065 to 0.164) and showed no significant additional effects on PU (H9, *β*=.030, *P*=.54, 95% CI −0.067 to 0.125) or PR (H10, *β*=−.042, *P*=.51, 95% CI −0.164 to 0.081). This contrasts with UTAUT, in which SI is theorized as a determinant of BI, although its strength is context-dependent and may vary according to individual and situational characteristics [9,12]. Several characteristics of the present case may have contributed to the weak SI effect. The sample was predominantly young and technically knowledgeable, actual ePA use was still uncommon, and SI was operationalized using a single item. Moreover, most respondents had limited practical experience with the ePA and may therefore not yet have been exposed to stable social norms regarding its use. Because the present study did not examine the full set of moderators proposed by UTAUT for SI, the nonsignificant finding should not be interpreted as evidence against the UTAUT relationship in general. Rather, it suggests that SI was not a salient determinant of ePA acceptance in this early-rollout and low-experience context. Replication after broader diffusion of the ePA and in more heterogeneous samples is therefore warranted.

Moreover, the indicators existing health problems (H19, *β*=−.089, *P*=.34, 95% CI −0.267 to 0.088), regular medication (H20, *β*=.060, *P*=.49, 95% CI −0.115 to 0.231), and support (H14, *β*=−.012, *P*=.88, 95% CI −0.165 to 0.139) produced no additional explanatory power. The nonsignificant influence of existing health problems and regular medication may be attributed to the predominantly young sample, in which health problems are less likely to occur. In addition, support may play a minor role for our respondents since only 9 of 149 reported low technical understanding. Technological affinity was not significantly associated with PU (H18, *β*=.094, *P*=.08, 95% CI −0.014 to 0.198). The hypothesis regarding the effect of technological affinity on PR was also not supported (H18, *β*=−.064, *P*=.29, 95% CI −0.185 to 0.055). Similarly, the hypothesized influence of technological affinity on PEOU was statistically insignificant (H18, *β*=−.092, *P*=.16, 95% CI −0.221 to 0.037). The assumed age (H16, *β*=−.056, *P*=.55, 95% CI −0.237 to 0.126) and gender (H17, *β*=.028, *P*=.75, 95% CI −0.142 to 0.198) moderation effects for PU→BI were also not significant. It is possible that existing moderation effects could not be detected due to the skewed and small sample.

In the present sample, we did not detect a statistically significant association between BI and self-reported actual ePA use. Only 35 participants reported actual ePA use, which limited the amount of information available for estimating this relationship. The nonsignificant estimate should therefore not be interpreted as evidence that behavioral intention is unrelated to actual ePA use in the broader population. Self-reported ePA use was low (35/149, 23.5%). BI was not significantly associated with actual use (H15, OR=1.37, *β*=.317, *P*=.15, 95% CI −0.113 to 0.748). Similar findings have also been reported in other studies [65]. At the time of data collection, the ePA had not yet been fully implemented, and participation in the system required a complicated onboarding process. This may additionally explain the low adoption rate. Even 1 year after full implementation, reports from health insurance providers indicate that only 3.6% of stored health records are actively used by patients [66].

It is therefore likely that many participants had limited or no practical experience with the system, and that some responses were based on hypothetical assumptions rather than actual usage. Future studies should distinguish more clearly between record availability, activation, occasional use, and repeated feature-specific use.

Finally, the open-ended qualitative responses supported the quantitative results and further highlighted what is specifically at stake in HDS acceptance. Participants most often mentioned centralized storage, emergency usefulness, and easier access to information as advantages, but they also expressed concerns about privacy breaches, cyberattacks, misuse of data, lack of trust in the technical infrastructure, and possible organizational problems in medical practice. These responses are consistent with broader literature showing that patient acceptance of health data sharing depends not only on individual benefit but also on perceived safeguards, institutional trustworthiness, and clarity of governance arrangements [4-7,17]. Together, the results are consistent with the view that HDS acceptance cannot be reduced to classical usability-and-benefit logic alone. Rather, it also depends on whether data practices and governance structures appear understandable, controllable, and trustworthy from the patient perspective.

Overall, the findings provide evidence for several individual relationships in the present sample and partial support for the selected ePA-specific model. They provide substantially weaker evidence regarding the broader validity of HDS-TAM as a general acceptance framework. However, the possible explanations for unsupported hypotheses are specific to our sample. They do not rule out that HDS-TAM may need refinement and adjustments, as it was only derived theoretically to combine predictors and constructs that appear to be relevant in HDS-related acceptance research. Accordingly, the comparatively large PU→BI path coefficient should not be interpreted as unequivocal evidence of a distinct structural relationship between the 2 constructs. Given their limited discriminant validity, the estimated magnitude of this association may partly reflect empirical overlap between PU and BI and should therefore be interpreted cautiously.

The present findings should be distinguished across 3 levels of inference. First, the study provides evidence for several individual hypothesized relationships within the present sample. Second, the findings provide partial empirical support for the selected ePA-specific instantiation of HDS-TAM, with 8 of the 20 original hypotheses supported. Third, the present case evaluation does not establish the validity or generalizability of HDS-TAM as an overarching acceptance framework. Evaluating the framework at this broader level requires prospective replication across independent samples, additional HDS applications, and different stakeholder groups.

### Limitations and Threats to Validity

#### Construct and Measurement Validity

Construct validity is affected by the high HTMT value of 0.981 observed between PU and BI, indicating limited discriminant validity according to established PLS-SEM guidelines [62]. However, inspection of the questionnaire items did not reveal substantial conceptual overlap. Given that PU is theorized as the primary antecedent of BI in both TAM and UTAUT, some degree of empirical overlap is not unexpected. Nevertheless, the strong association suggests that the magnitude of the corresponding path coefficient must be interpreted cautiously.

Several constructs were measured using a small number of indicators, and some exploratory constructs relied on single-item measures, limiting their ability to capture the full complexity of the underlying concepts. All measurements were self-reported and collected within the same survey instrument, which may introduce common method bias despite the observed variation in effect sizes and the presence of multiple unsupported hypotheses. Although respondents could skip individual items, the forced-choice 4-point response format may have influenced response distributions and limits direct comparability with studies using 5- or 7-point Likert scales or a neutral midpoint.

The original reflective operationalizations of PN and T showed poor internal consistency and did not exhibit stable unidimensional structures in the exploratory measurement analysis. This prompted a post hoc, theory-guided respecification of the retained indicators as composites. Although this specification is conceptually plausible, it was not defined a priori and was developed using the same sample in which the structural model was subsequently estimated. The composite specification should therefore be considered preliminary and requires prospective evaluation in an independent sample. PN was measured with 2 items, and T with 5 items. For T, informational clarity about data processing may differ conceptually from control over research use or expectations of notification. Similarly, PN may vary depending on whether it refers to acute health-related necessity, record management, or broader care coordination. These findings do not argue against the relevance of transparency or need. Rather, in future HDS-TAM studies, the item statements should be sharpened and differentiated to better match the constructs, and more items should be used for operationalization [13,26,35,36]. These theory-driven arguments led to the decision to model T and PN as composite rather than reflective constructs. Regarding self-reported ePA usage, the questionnaire did not capture detailed information about actual system interaction, such as which specific functionalities were used.

Exploratory measurement evaluation, item refinement, and subsequent PLS-SEM estimation were conducted using the same sample. Consequently, the resulting measurement specification may partly reflect sample-specific characteristics, and the retained item structure should not be interpreted as independently confirmed. Future studies should prospectively evaluate the revised measurement model in an independent sample.

Some items were excluded from the PLS-SEM estimation, which may additionally weaken the overall conclusions on the HDS-TAM model.

#### Internal Validity

The cross-sectional design limits the ability to draw causal conclusions. Although the structural relationships were derived from established acceptance theories, temporal precedence cannot be verified empirically. Moreover, data collection took place during the early public rollout of the ePA under an opt-in regime, when many respondents had limited direct experience with the system. Self-reported actual use of the ePA was low (35/149, 23.5%). Because only 35 participants reported actual ePA use, conclusions regarding use-related mechanisms are based on limited empirical information and should be regarded as exploratory. Consequently, participants may have evaluated anticipated rather than experienced usefulness, ease of use, and risks. The lack of a significant relationship between BI and actual ePA use further suggests that relevant determinants of adoption behavior may not have been captured. Similar intention-use mismatches have been reported in health behavior research [67].

#### External Validity

The sample in this study was imbalanced: 59.7% (n=89) of participants identified as female, 33.6% (n=50) as male, 0.7% (n=1) as diverse, and 6% (n=9) did not provide a response. Furthermore, 131 participants (87.9%) were university students (n=80) or employees (n=51), and 67.8% (n=101) were younger than 30 years. The imbalance can be attributed to the recruitment strategy that initially addressed primarily students and university members as a convenience sample. Recruitment was conducted via university mailing lists, social media, and acquaintances/network sharing. The demographic imbalances limit the generalizability of the findings regarding acceptance of the ePA. The sample does not adequately represent the broader German population. Accordingly, the observed associations should be interpreted as sample-specific evidence from this convenience sample rather than as estimates that can be generalized to German patients, the general population, or the broader population of actual ePA users.

#### Conclusion Validity

A subset of HDS-TAM was used as the hypothesis-testing model for a patient-centric survey. The regression-based sensitivity analysis indicated that the available sample provided reasonable sensitivity for medium effects but limited sensitivity for small effects. Accordingly, small structural relationships and interaction effects may have remained undetected. However, the lack of support for several hypotheses is unlikely to be explained by statistical power alone, as many of the corresponding relationships were associated with negligible effect sizes. The sample was skewed toward younger respondents, potentially reducing variability in health-related and adoption-relevant characteristics. Finally, data were collected during the early opt-in phase of the ePA, when actual use was still uncommon, meaning that respondents’ evaluations likely reflected expectations rather than real experience. Consequently, in the present survey, HDS-TAM appears better suited to explaining intention formation than actual adoption behavior, and conclusions regarding use-related mechanisms should be interpreted cautiously. No separate a priori power analysis was conducted for the binary actual-use outcome. Although the logistic regression included only 1 predictor, only 35 actual-use events were observed, resulting in limited statistical information and reduced precision for estimating the BI-ASU association. Accordingly, the nonsignificant result should be interpreted as inconclusive rather than as evidence of no association.

### Conclusions and Future Work

We developed HDS-TAM as an HDS-oriented framework for analyzing technology acceptance in contexts where system use is closely linked to questions of governance, control, transparency, and data sharing. In the patient-focused case evaluation of the ePA, PU showed the largest positive association with behavioral intention, whereas PR showed a smaller negative association. The comparatively large PU-BI association should be interpreted cautiously because discriminant validity between the 2 constructs was limited. PEOU showed primarily indirect associations with behavioral intention through usefulness and risk, whereas PN was primarily indirectly associated with behavioral intention through usefulness.

The study also showed that some conceptually important constructs, especially transparency and PN, may require more differentiated operationalization in HDS contexts. The present findings primarily support individual relationships observed in this sample and provide only partial evidence for the selected ePA-specific model; they should not be interpreted as validation of HDS-TAM as a generalizable framework. HDS-TAM should therefore not be regarded as a fully validated model, but as a promising framework that helps identify which factors are most relevant for acceptance in HDS contexts and where further conceptual refinement is needed.

Future research should further refine HDS-TAM and evaluate it in larger and more heterogeneous samples, allowing for more reliable estimation of small effects and subgroup differences. Replication of the survey after broader diffusion of the ePA may provide a more suitable context for investigating the relationship between behavioral intention and actual ePA use. To address the observed discriminant validity concerns, future studies should refine the operationalization of PU and behavioral intention. In addition, longitudinal surveys could better separate belief formation from usage intentions. Revised and broader item sets, for example, for transparency and PN, are required. Incorporating implementation-related factors such as facilitating conditions, trust, digital health literacy, physician endorsement, and objective usage measures may improve the explanation of actual adoption behavior and help account for the observed difference between intention scores and limited actual use. Finally, only a patient-oriented subset of HDS-TAM was used in the survey. It would be advisable to expand the validation to include other HDS applications and stakeholders.

## Supplementary material

10.2196/99366 Multimedia Appendix 1 Electronic patient record case evaluation study: list of hypotheses.

10.2196/99366 Multimedia Appendix 2 Electronic patient record case evaluation study: constructs and associated items.

10.2196/99366 Multimedia Appendix 3 Electronic patient record case evaluation study: questionnaire (together with English translation).

10.2196/99366 Multimedia Appendix 4 Electronic patient record case evaluation study: sample characteristics.

10.2196/99366 Multimedia Appendix 5 Electronic patient record case evaluation survey: missing data details.

10.2196/99366 Multimedia Appendix 6 Electronic patient record case evaluation study: answers to open questions (together with English translation).

10.2196/99366 Multimedia Appendix 7 CHERRIES checklist.

## Abbreviations

ASU: actual system use
AVE: average variance extracted
BI: behavioral intention to use
EFA: exploratory factor analysis
EHDS: European Health Dataspace
ePA: elektronische patientenakte (German electronic patient record)
H-TAM: health care technology acceptance model
HDS: health dataspace
HDS-TAM: health dataspace technology acceptance model
HTMT: heterotrait-monotrait ratio of correlations
KMO: Kaiser-Meyer-Olkin (criterion)
MAR: missing at random
MCAR: missing completely at random
mHealth: mobile health
MICE: Multiple Imputation by Chained Equations
MNAR: missing not at random
PEOU: perceived ease of use
PLS-SEM: partial least squares structural equation modeling
PN: perceived need
PR: perceived risk
PU: perceived usefulness
RQ: research question
RR: Rubin rules
SI: social influence
TAM: Technology Acceptance Model
TAM2: Technology Acceptance Model version 2
TAM3: Technology Acceptance Model version 3
UB: use behavior
UTAUT: Unified Theory of Acceptance and Use of Technology
UTAUT2: Unified Theory of Acceptance and Use of Technology version 2
VIF: variance inflation factor
ρC: composite reliability

## References

[1] (2025). Regulation (EU) 2025/327 of the European Parliament and of the Council of 11 February 2025 on the European Health Data Space and amending Directive 2011/24/EU and Regulation (EU) 2024/2847 (text with EEA relevance). European Union.

[2] Kympouropoulos S (2023). Real world evidence: methodological issues and opportunities from the European Health Data Space. BMC Med Res Methodol.

[3] Otto B, ten Hompel M, Wrobel S (2022). Designing Data Spaces: The Ecosystem Approach to Competitive Advantage.

[4] Cascini F, Pantovic A, Al-Ajlouni YA, Puleo V, De Maio L, Ricciardi W (2024). Health data sharing attitudes towards primary and secondary use of data: a systematic review. eClinicalMedicine.

[5] Baines R, Stevens S, Austin D (2024). Patient and public willingness to share personal health data for third-party or secondary uses: systematic review. J Med Internet Res.

[6] de la Cruz PC, Lalova-Spinks T, Shabani M (2025). The European Health Data Space: an opportunity to strengthen citizen rights and engage citizens in health data governance. Front Med.

[7] Muller SHA, Kalkman S, van Thiel GJMW, Mostert M, van Delden JJM (2021). The social licence for data-intensive health research: towards co-creation, public value and trust. BMC Med Ethics.

[8] Davis FD (1989). Perceived usefulness, perceived ease of use, and user acceptance of information technology. MIS Q.

[9] Venkatesh V, Morris MG, Davis GB, Davis FD (2003). User acceptance of information technology: toward a unified view. MIS Q.

[10] Rahimi B, Nadri H, Afshar HL, Timpka T (2018). A systematic review of the technology acceptance model in health informatics. Appl Clin Inform.

[11] Holden RJ, Karsh BT (2010). The technology acceptance model: its past and its future in health care. J Biomed Inform.

[12] Blut M, Chong AYL, Tsigna Z (2022). Meta-analysis of the Unified Theory of Acceptance and Use of Technology (UTAUT): challenging its validity and charting a research agenda in the red ocean. JAIS.

[13] Harris MT, Rogers WA (2023). Developing a healthcare technology acceptance model (H-TAM) for older adults with hypertension. Ageing Soc.

[14] Thim C (2017). Technology acceptance in organisations [Doctoral Thesis in German]. https://nbn-resolving.org/urn:nbn:de:kobv:517-opus4-401070.

[15] Hu WF, Wu SZ, Qian Q (2025). Data spaces in medicine and health: technologies, applications, and challenges. Chin Med Sci J.

[16] Fröhlich H, Funck Hansen A, Hilvo M (2025). Reality check: the aspirations of the European Health Data Space amidst challenges in decentralized data analysis. J Med Internet Res.

[17] Olsen Q, Dyda A, Woods L (2025). Worldwide willingness to share health data high but privacy, consent and transparency paramount, a meta-analysis. NPJ Digit Med.

[18] AlQudah AA, Al-Emran M, Shaalan K (2021). Technology acceptance in healthcare: a systematic review. Appl Sci.

[19] Benbasat I, Barki H (2007). Quo vadis TAM?. JAIS.

[20] Venkatesh V, Davis FD (1996). A model of the antecedents of perceived ease of use: development and test. Decis Sci.

[21] Venkatesh V, Davis FD (2000). A theoretical extension of the Technology Acceptance Model: four longitudinal field studies. Manage Sci.

[22] Venkatesh V, Bala H (2008). Technology Acceptance Model 3 and a research agenda on interventions. Decis Sci.

[23] Venkatesh V, Thong JYL, Xu X (2012). Consumer acceptance and use of information technology: extending the Unified Theory of Acceptance and Use of Technology. MIS Q.

[24] Schretzlmaier P, Hecker A, Ammenwerth E (2022). Extension of the Unified Theory of Acceptance and Use of Technology 2 model for predicting mHealth acceptance using diabetes as an example: a cross-sectional validation study. BMJ Health Care Inform.

[25] Schomakers EM, Lidynia C, Vervier LS, Valdez AC, Ziefle M (2022). Applying an extended UTAUT2 model to explain user acceptance of lifestyle and therapy mobile health apps: survey study. JMIR mHealth uHealth.

[26] Safi S, Danzer G, Raha S, Nassar E, Hufert FT, Schmailzl KJG (2021). Does being ill improve acceptance of medical technology?—A patient survey with the technology usage inventory. Int J Environ Res Public Health.

[27] (2022). Satzung der kommission für ethik und doppelverwendungsfähige forschung des senats der Universität Koblenz vom 6 [Article in German]. University of Koblenz.

[28] (2023). Satzung zur sicherung guter wissenschaftlicher praxis an der Universität Koblenz [Article in German]. University of Koblenz.

[29] (2007). Guidelines for performing systematic literature reviews in software engineering. https://legacyfileshare.elsevier.com/promis_misc/525444systematicreviewsguide.pdf.

[30] Lo A, Jenkins PH, Choobineh J (2019). Patient’s acceptance of IT-assisted self-monitoring: a multiple-case study. J Comput Inform Syst.

[31] Park I, Kim D, Moon JS, Kim S, Kang Y, Bae S (2022). Searching for new technology acceptance model under social context: analyzing the determinants of acceptance of intelligent information technology in digital transformation and implications for the requisites of digital sustainability. Sustainability.

[32] Wang H, Zhang J, Luximon Y, Qin M, Geng P, Tao D (2022). The determinants of user acceptance of mobile medical platforms: an investigation integrating the TPB, TAM, and patient-centered factors. Int J Environ Res Public Health.

[33] Aljarboa S, Miah SJ Investigating acceptance factors of clinical decision support systems in a developing country context.

[34] Taneja B, Bharti K (2025). Role of behavioural abstraction (vs concrete) levels in accepting health technologies. Global Knowl, Memory Commun.

[35] Czaja S, Beach S, Charness N, Schulz R (2013). Technologies for Active Aging.

[36] Lorenzen-Huber L, Boutain M, Camp LJ, Shankar K, Connelly KH (2011). Privacy, technology, and aging: a proposed framework. Ageing Int.

[37] Wilkowska W, Heek JO, Brauner P, Ziefle M Wind of change? Attitudes towards aging and use of medical technology.

[38] Schretzlmaier P, Hecker A, Ammenwerth E (2023). Predicting mHealth acceptance using the UTAUT2 technology acceptance model: a mixed-methods approach. Stud Health Technol Inform.

[39] Oh K, Kho HS, Choi Y, Lee S (2022). Determinants for successful digital transformation. Sustainability.

[40] Duzenli O, Felekoglu B, Tasan AS Factors affecting the adoption of technological service innovations.

[41] Jacob C, Sanchez-Vazquez A, Ivory C (2020). Social, organizational, and technological factors impacting clinicians’ adoption of mobile health tools: systematic literature review. JMIR mHealth uHealth.

[42] Kang JW, Namkung Y (2019). The role of personalization on continuance intention in food service mobile apps. IJCHM.

[43] Ahlan AR, Ahmad BI (2022). An overview of patient acceptance of health information technology in developing countries: a review and conceptual model. IJISPM.

[44] Shin DH (2007). User acceptance of mobile internet: implication for convergence technologies. Interact Comput.

[45] Dhagarra D, Goswami M, Kumar G (2020). Impact of trust and privacy concerns on technology acceptance in healthcare: an Indian perspective. Int J Med Inform.

[46] Pai RR, Alathur S (2019). Predicting mobile health technology acceptance by the Indian rural community. Int J Electron Gov Res.

[47] Cheng YM (2015). Towards an understanding of the factors affecting m-learning acceptance: roles of technological characteristics and compatibility. Asia Pacific Manage Rev.

[48] Arkorful VE, Shuliang Z, Muhideen S, Basiru I, Hammond A (2020). An empirical investigation of health practitioners technology adoption: the mediating role of electronic health. Int J Public Adm.

[49] Marcus AA (1981). Policy uncertainty and technological innovation. Acad Manage Rev.

[50] Bruhn CM (2007). Enhancing consumer acceptance of new processing technologies. Innovative Food Sci Emerging Technol.

[51] Lu Z, Cui T, Tong Y, Wang W (2020). Examining the effects of social influence in pre-adoption phase and initial post-adoption phase in the healthcare context. Inform Manage.

[52] Roumani Y, Nwankpa JK, Roumani YF (2015). The impact of incentives on the intention to try a new technology. Technol Anal Strategic Manage.

[53] Bozan K, Mooney D (2020). Delivering Superior Health and Wellness Management with IoT and Analytics.

[54] Molino M, Cortese CG, Ghislieri C (2021). Technology acceptance and leadership 4.0: a quali-quantitative study. Int J Environ Res Public Health.

[55] Stellmach C, Muzoora MR, Thun S (2022). Digitalization of health data: interoperability of the proposed European health data space. Stud Health Technol Inform.

[56] Rise J, Sheeran P, Hukkelberg S (2010). The role of self‐identity in the theory of planned behavior: a meta‐analysis. J Appl Soc Psychol.

[57] Die elektronische patientenakte (epa) für alle [Article in German]. Bundesministerium für gesundheit (BMG).

[58] Kankaraš M, Capecchi S (2025). Neither agree nor disagree: use and misuse of the neutral response category in Likert-type scales. METRON.

[59] Shrestha N (2021). Factor analysis as a tool for survey analysis. Am J Appl Math Stat.

[60] Hill BD (2011). The sequential Kaiser-Meyer-Olkin procedure as an alternative for determining the number of factors in common-factor analysis: a Monte Carlo simulation. https://openresearch.okstate.edu/server/api/core/bitstreams/6a1d5d61-1d2a-4c7c-bf91-9fa6a89bf95a/content.

[61] King WR, He J (2006). A meta-analysis of the technology acceptance model. Inform Manage.

[62] Henseler J, Ringle CM, Sarstedt M (2015). A new criterion for assessing discriminant validity in variance-based structural equation modeling. J Acad Mark Sci.

[63] Andrews L, Gajanayake R, Sahama T (2014). The Australian general public’s perceptions of having a personally controlled electronic health record (PCEHR). Int J Med Inform.

[64] Liu CF, Tsai YC, Jang FL (2013). Patients’ acceptance towards a web-based personal health record system: an empirical study in Taiwan. Int J Environ Res Public Health.

[65] Webb TL, Sheeran P (2006). Does changing behavioral intentions engender behavior change? A meta-analysis of the experimental evidence. Psychol Bull.

[66] (2026). Digitalisierung: zahl der aktiven epa-nutzer bleibt gering [Article in German]. Pharmazeutische Zeitung.

[67] Yeo Y, Sohn C (2021). Intention-behavior gap in the use of personal health records (PHRs): investigation of social determinants. Int J E-Adoption.

[68] Vasileva V, Wolf E, Riediger V, Ahmadian AS, Buschmann D, Jürjens J (2026). HDS-TAM ePA case study - raw survey answers from SoSci Survey dataset.

