# Supplementary material for: Health Dataspace Technology Acceptance Model (HDS-TAM): Development and Case Evaluation Study of Patient Acceptance of Germany’s Electronic Patient Record
Source: JMIR Med Inform. 2026 Nov 2;14:e99366. doi: 10.2196/99366 (PMC13726799; doi:10.2196/99366)
Supplement: Supplementary file 1 [file medinform-v14-e99366-s001.pdf]

HDS-TAM ePA case evaluation: hypotheses model

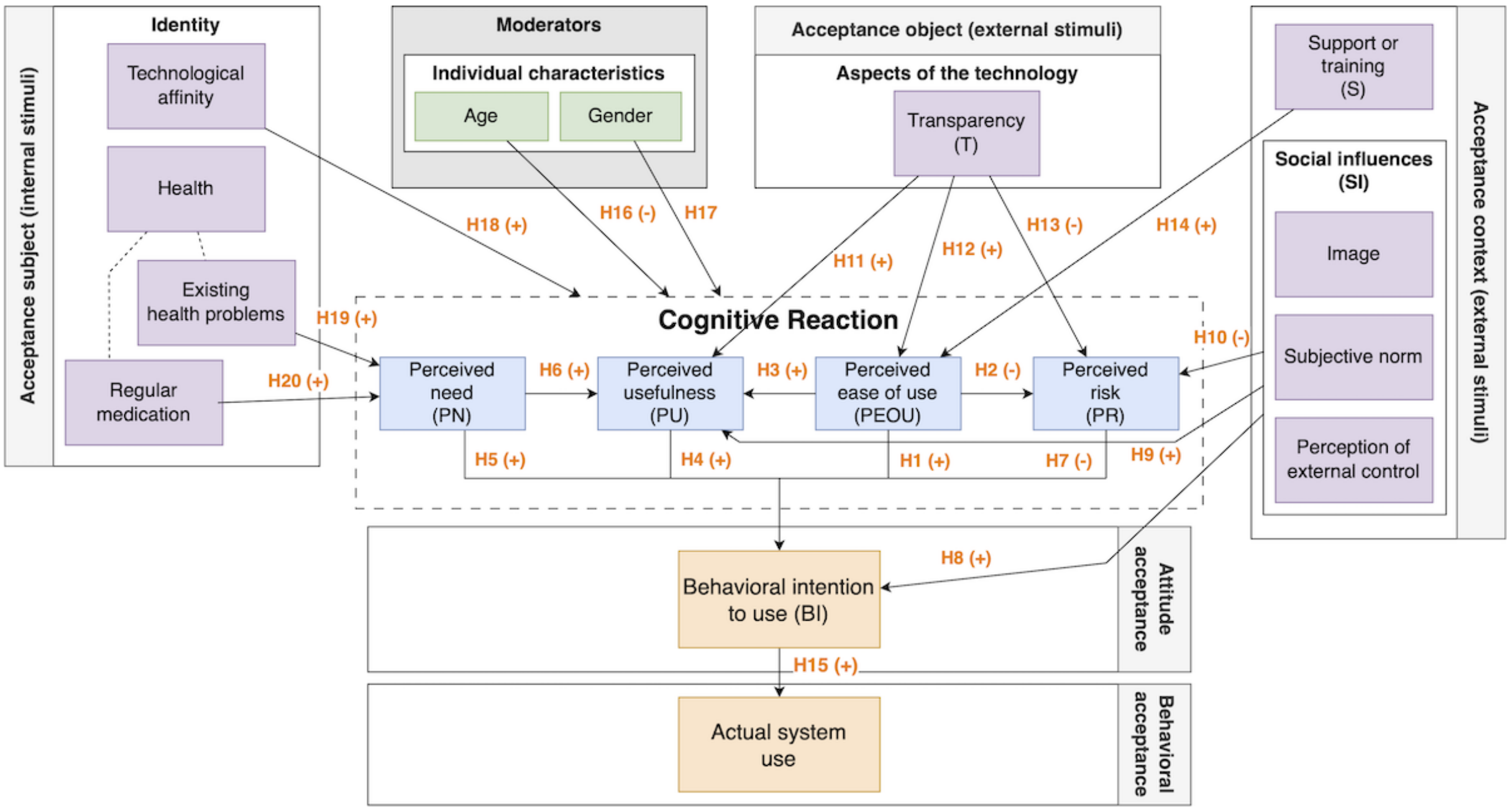

HDS-TAM ePA case evaluation: list of hypotheses

No.	Constructs Influence	Direction of Correlation
H1	PEOU → BI	positive
H2	PEOU → PR	negative
H3	PEOU → PU	positive
H4	PU → BI	positive
H5	PN → BI	positive
H6	PN → PU	positive
H7	PR → BI	negative
H8	SI → BI	positive
H9	SI → PU	positive
H10	SI → PR	negative
H11	TR → PU	positive
H12	TR → PEOU	positive
H13	TR → PR	negative
H14	SUP → PEOU	positive
H15	BI → ASU	positive
	Moderator Effects	*Influence*
H16	Age moderates PN, PU, PEOU, PR, SI → BI	negative
H17	Gender moderates PN, PU, PEOU, PR, SI → BI	–
	Other variables	*Influence*
H18	Technological affinity → PU, PR, PEOU	positive
H19	Existing health problems → PN	positive
H20	Regular medication → PN	positive
