# Supplementary material for: Health Dataspace Technology Acceptance Model (HDS-TAM): Development and Case Evaluation Study of Patient Acceptance of Germany’s Electronic Patient Record
Source: JMIR Med Inform. 2026 Nov 2;14:e99366. doi: 10.2196/99366 (PMC13726799; doi:10.2196/99366)
Supplement: Supplementary file 2 [file medinform-v14-e99366-s002.pdf]

HDS-TAM ePA Case Evaluation: Constructs and Associated items.

Responses to items with suffix „i“ were inverted for the analysis.

Items in braces were excluded from PLS-SEM estimation.

Construct	Item	Statement
Behavioural Intention	BI1	I plan to use the ePA regularly to manage my medications.
	BI2	I would also use the ePA in unexpected situations, such as an emergency.
	BI3	I can imagine using the ePA for video consultations with my doctor.
	(BI4)	I am willing to share my pseudonymized ePA data for medical research purposes.
	(BI5)	The possibility that my data could contribute to the development of new treatments motivates me to share my data.
Perceived Ease of Use	PEOU1	Using the ePA is easy and straightforward for me.
	PEOU2	I find it easy to understand the features of the ePA.
Perceived Need	PN1	It’s important to me to be able to review diagnoses and medical records before seeing a doctor so I can better prepare for the appointment.
	PN2	I feel that the ePA meets a genuine need for the storage of my health data.
Perceived Risk	PR1i	The ePA helps me avoid misunderstandings regarding prescriptions and identify drug interactions.
	(PR2)	I’m afraid that I can’t control which medications my doctor can see.
	(PR3)	I’m afraid I don’t know how to manage access rights in the ePA.
	(PR4i)	I feel more secure when I can use the ePA app to specify who is allowed to view which data.
	PR5	I'm afraid of losing control over my health data.
	PR6i	I have confidence in the security and privacy of my health data in the ePA.
	PR7	I have a feeling that my data might be misused.
	PR8i	Storing data in accordance with the highest security standards increases my trust and helps prevent the loss of important medical records.
	(PR9i)	I am confident that doctors can only access the electronic health record with my consent.
	(PR10)	I think it’s important that I can control which other digital apps are allowed to save data to my ePA.
	PR11	I am concerned that my health data could be misused despite pseudonymization.
	PR12	I have no confidence in the security and privacy of my health data in the ePA.

HDS-TAM ePA Case Evaluation: Constructs and Associated Items (continued)

Responses to items with suffix „i“ were inverted for the analysis.

Items in braces were excluded from PLS-SEM estimation.

Construct	Item	Statement
Perceived Usefulness	PU1	With the ePA, I find it easier to ask specific questions when talking to doctors.
	PU2	It’s important to me to be able to centrally store and share health records suchas doctor's notes and medical records.
	PU3	Using the ePA saves me time when managing my health information.
	PU4	Thanks to the ePA, I was able to avoid duplicate examinations.
	(PU5)	Even with the ePA, I think it makes sense to have examinations repeated, even if previous results are available.
	PU6	Using the ePA makes it easier for me to keep track of my medications.
	PU7	I find it helpful that my medications are automatically stored in a digital overview.
	PU8	I find it useful that doctors can access my previous diagnoses and treatments in emergencies.
	PU9	Using the ePA reduces the risk of incorrect treatment in emergencies.
	PU10	The more I learn about the ePA, the more useful it seems to me for my healthcare.
	PU11	Using the ePA improves my overall experience with the healthcare system.
Support	S1	The ability to set up the settings together with a representative from the health insurance company is important to me.
Social Influence	SI1	The influence of doctors, family, and friends played a role in my decision to use the ePA.
Transparency	(T1i)	I have no knowledge of the risks that may arise from using the ePA.
	T2	I have received sufficient information about data processing in the ePA.
	(T3)	It is important to me to decide for myself whether my ePA data may be used for research.
	T4	I trust that my data will only be used for scientific purposes.
	T5	It is important to me that I receive a notification every time my data is used for research purposes.
