# Supplementary material for: Health Dataspace Technology Acceptance Model (HDS-TAM): Development and Case Evaluation Study of Patient Acceptance of Germany’s Electronic Patient Record
Source: JMIR Med Inform. 2026 Nov 2;14:e99366. doi: 10.2196/99366 (PMC13726799; doi:10.2196/99366)
Supplement: Supplementary file 3 [file medinform-v14-e99366-s003.pdf]

Vorschau des Fragebogens "Fragebogen"

Remark: Item labels were added to this questionnaire preview. The wording of item PR12 in Scenario 8 was edited to match the fielded version. The fielded version is also documented in the variable labels of the deposited SPSS dataset.

0% ausgefüllt

## Umfrage zur Untersuchung der Akzeptanz und Transparenz von medizinischen Datenräumen und deren Technologien am Beispiel der elektronischen Patientenakte

Sehr geehrte/r Teilnehmer/in,

vielen Dank für Ihr Interesse an unserer wissenschaftlichen Befragung! Die Befragung findet im am Institut für Softwaretechnik des Fachbereichs 4: Informatik der Universität Koblenz statt.

Das Ziel der Befragung ist es, Informationen über die Akzeptanz und Transparenz von medizinischen Datenräumen und deren Softwarelösungen zu erlangen. Ein Beispiel dafür ist die elektronische Patientenakte, kurz ePA. Diese wird als Beispiel für die Befragung genutzt.

Ihre Teilnahme an der Studie ist freiwillig. Sie können Ihre Teilnahme jederzeit ohne die Angabe von Gründen abbrechen, ohne dass Ihnen daraus Nachteile entstehen. Bitte beantworten Sie die Fragen der Reihe nach und so genau und ehrlich wie möglich. Die vollständige Beantwortung dauert ca. 15 Minuten. Bitte nutzen Sie zum Navigieren den "Weiter" und "Zurück" Button am Seitenende.

Die Speicherung der Daten erfolgt unter strengsten Bestimmungen der Geheimhaltung. Das heißt es ist niemandem möglich, Ihre Daten mit Ihrer Person in Verbindung zu bringen.

Aus Gründen der besseren Lesbarkeit wird auf die gleichzeitige Verwendung der Sprachformen männlich, weiblich und divers (m/w/d) verzichtet. Sämtliche Personenbezeichnungen gelten gleichermaßen für alle Geschlechter.

Survey on the Acceptance and Transparency of Medical Data Spaces and Their Technologies: The Case of the Electronic Health Record

Dear Participant,
Thank you very much for your interest in our research survey! The survey is being conducted at the Institute for Software Engineering in Department 4: Computer Science at the University of Koblenz.

The aim of the survey is to gather information on the acceptance and transparency of medical data spaces and their software solutions. One example of this is the electronic health record, or eHR for short. This is being used as an example for the survey.

Your participation in the study is voluntary. You may withdraw your participation at any time without providing a reason and without incurring any disadvantages. Please answer the questions in order and as accurately and honestly as possible. It will take approximately 15 minutes to complete the survey. Please use the “Next” and “Back” buttons at the bottom of the page to navigate.

Data is stored under the strictest confidentiality regulations. This means that no one can link your data to your identity.

For the sake of readability, we have omitted the simultaneous use of gendered forms (male, female, and diverse; m/f/d). All references to persons apply equally to all genders.

Weiter

Institute for Software Technology, University of Koblenz – 2025

Vorschau des Fragebogens "Fragebogen"

university of koblenz
Computer Science

8% ausgefüllt

**Die Richtlinien guter ethischer Forschung sehen vor, dass sich die Teilnehmer/innen an empirischen Studien explizit und nachvollziehbar mit der Teilnahme einverstanden erklären.**

**Freiwilligkeit.** Ihre Teilnahme an dieser Untersuchung ist freiwillig. Es steht Ihnen zu jedem Zeitpunkt dieser Studie frei, Ihre Teilnahme abzubrechen, ohne dass Ihnen daraus Nachteile entstehen.

**Anonymität.** Ihre Daten sind selbstverständlich vertraulich, werden nur in anonymisierter Form ausgewertet und nicht an Dritte weitergegeben. Demographische Angaben wie Alter oder Geschlecht lassen keinen eindeutigen Schluss auf Ihre Person zu.

**Fragen.** Falls Sie noch Fragen zu dieser Studie haben sollten, finden Sie im Anschluss ein Impressum mit Kontaktdaten der Studienleiter.

Hiermit bestätige ich, dass ich mindestens 18 Jahre alt bin sowie die Einverständniserklärung gelesen und verstanden habe.

Consent

- ◯ Nein (nicht an der Studie teilnehmen)
- ◯ Ja

Zurück Weiter

The guidelines for sound ethical research require that participants in empirical studies explicitly and clearly consent to their participation.

Voluntary Participation. Your oarticipation in this study is voluntary. You are free to withdraw from the study at any time without suffering any disadvantages as a result.

Anonymity. Your data will, of course, be treated confidentially, will only be analyzed in anonymized form, and will not be shared with third parties. Demographic information such as age or gender does not allow for any clear identification of you as an individual.

Questions. If you have any questions about this study, you will find an imprint with the contact information of the study leaders below.

I hereby confirm that I am at least 18 years old and that I have read and understood the consent form.

O No (do not participate in the study)
O Yes

Institute for Software Technology, University of Koblenz – 2025

Vorschau des Fragebogens "Fragebogen"

15% ausgefüllt

Gender

**1. Zunächst möchten wir Sie bitten, einige Angaben zu Ihrer Person zu machen.**

Sie sind: [Bitte auswählen]

AgeGroup

**2. Wie alt sind Sie?**

[Bitte auswählen]

EducationLevel

**3. Welchen Bildungsabschluss haben Sie?**

Bitte wählen Sie den höchsten Bildungsabschluss, den Sie bisher erreicht haben.

- ◯ Schule beendet ohne Abschluss
- ◯ Noch Schüler
- ◯ Hauptschulabschluss
- ◯ Mittlere Reife, Realschul- oder gleichwertiger Abschluss
- ◯ Abgeschlossene Lehre
- ◯ Fachabitur, Fachhochschulreife
- ◯ Abitur, Hochschulreife
- ◯ Fachhochschul-/Hochschulabschluss
- ◯ Anderer Abschluss, und zwar: EducationLevelOther

Occupation

**4. Was machen Sie beruflich?**

- ◯ Schüler/in
- ◯ In Ausbildung
- ◯ Student/in
- ◯ Angestellte/r
- ◯ Beamte/r
- ◯ Selbstständig
- ◯ Arbeitslos/Arbeit suchend
- ◯ Sonstiges: OccupationOther

1. First, we would like to ask you to provide some personal information.

You are: [Please select]
- male
- female
- diverse

2. How old are you? [Please select]
- younger than 15 years
- 15-19 years
- 20-24 years
- 25-29 years
- 30-34 years
- 35-39 years
- 40-44 years
- 45-50 years
- 50-54 years
- 55-59 years
- 60-64 years
- 65 years or older

3. What is your highest level of education? Please select the highest level of education you have attained so far.

O Completed school without a diploma
O School student
O Elementary school diploma
O Middle school diploma, secondary school diploma, or equivalent
O Completed apprenticeship
O Vocational diploma, technical college entrance qualification
O High school diploma, university entrance qualification
O Technical college/university degree
O Other degree, specifically: …

4. What is your occupation?

O School Student
O Apprenticeship
O University Student
O Employee
O Civil servant
O Self-employed
O Unemployed/job seeker
Other: …

TechnicalUnderstanding

**5. Wie schätzen Sie Ihre allgemeinen technischen Kenntnisse ein?**

- ◯ sehr hoch
- ◯ hoch
- ◯ durchschnittlich
- ◯ gering
- ◯ sehr gering

5. How would you rate your general technical knowledge?

O very high
O high
O average
O low
O very low

ActualEPAUse

**6. Nutzen Sie bereits die elektronische Patientenakte?**

- ◯ ja
- ◯ nein

6. Do you already use the electronic health record?

O yes
O no

RegularMedication

**7. Nehmen Sie regelmäßig Medikamente zu sich?**

- ◯ ja
- ◯ nein

7. Do you take medication regularly?

O yes
O no

HealthIssues

**8. Haben Sie Vorerkrankungen?**

- ◯ ja
- ◯ nein

8. Do you have any pre-existing health issues?

O yes
O no

Zurück Weiter

Institute for Software Technology, University of Koblenz – 2025

university of koblenz
Computer Science

23% ausgefüllt

## Informationen zur ePA

**Bitte lesen Sie die nachfolgenden Informationen aufmerksam durch.**

Seit dem 15. Januar 2025 steht jedem gesetzlich Versicherten die elektronische Patientenakte zur Verfügung. Die elektronische Patientenakte (ePA) ist ein digitales System, das Ihnen als Patient dabei hilft, Ihre Gesundheitsinformationen zentral und sicher zu speichern und zu verwalten. Sie dient zur Verbesserung der Qualität und Effizienz der Gesundheitsversorgung. Die Nutzung der ePA ist freiwillig und kostenlos. Zugang zur ePA erhalten Sie in der jeweiligen App Ihrer Krankenkasse.

Die ePA soll Prozesse im Gesundheitswesen vereinfachen, den Informationsaustausch verbessern und dabei die sensiblen Daten schützen. Die folgenden Beschreibungen zeigen typische Alltagssituationen, in denen die ePA genutzt wird. Anschließend werden dazu Aussagen getroffen, bei welchen Sie entscheiden sollen, ob Sie diesen zustimmen.

Information About the ePA

Please read the following information carefully. Since January 15, 2025, the electronic patient record has been available to everyone with statutory health insurance. The electronic patient record (ePA) is a digital system that helps you, as a patient, to store and manage your health information in a centralized and secure manner. It serves to improve the quality and efficiency of healthcare. Use of the ePA is voluntary and free of charge. You can access the ePA through your health insurance provider’s app.

The ePA is designed to simplify processes in the healthcare system, improve the exchange of information, and protect sensitive data. The following descriptions illustrate typical everyday situations in which the ePA is used. Afterward, statements are provided regarding these situations, and you will be asked to decide whether you agree to them.

Zurück Weiter

university of koblenz
Computer Science

31% ausgefüllt

**Szenario 1: Übersicht über Diagnosen, Befunde und Speicherung von Dokumenten**

Scenario 1: Overview of Diagnoses, Test Results, and Document Storage

Sie haben wiederholt Arztbesuche oder Krankenhausaufenthalte hinter sich und mussten bisher Diagnosen, Befunde und Arztbriefe in Papierform oder in verschiedenen E-Mails verwalten. Das machte es schwierig, den Überblick zu behalten und sich auf Arzttermine vorzubereiten.

You have had multiple doctor's visits or hospital stays and have had to manage diagnoses, test results, and doctor's notes in paper form or across various emails up until now. This made it difficult to keep track of everything and prepare for doctor's appointments.

Mit der Nutzung der elektronischen Patientenakte (ePA) können Sie nun alle relevanten Gesundheitsinformationen an einem zentralen Ort speichern. Dadurch fällt es Ihnen leichter gezielte Fragen zu stellen und wichtige Dokumente mit Ihren Ärzten zu teilen. Ihr Arzt kann schneller auf Ihre vollständige Krankengeschichte zugreifen, sodass Doppeluntersuchungen vermieden werden können.

By using the electronic patient record (ePA), you can now store all relevant health information in one central location. This makes it easier for you to ask specific questions and share important documents with your doctors. Your doctor can access your complete medical history more quickly, so that duplicate tests can be avoided.

(All question items share the same 4 options)

| | Stimme gar nicht zu | Stimme eher nicht zu | Stimme eher zu | Stimme voll und ganz zu |
|---|---|---|---|---|
| | Strongly Disagree | Disagree | Agree | Strongly Agree |
| PN1 Es ist mir wichtig, Diagnosen und Befunde vor einem Arztbesuch durchlesen zu können, um mich besser auf den Termin vorzubereiten. / It's important to me to be able to review diagnoses and medical records before seeing a doctor so I can better prepare for the appointment. | ○ | ○ | ○ | ○ |
| PU1 Mit der ePA fällt es mir leichter, gezielte Fragen im Gespräch mit Ärzten zu stellen. / With the ePA, I find it easier to ask specific questions when talking to doctors. | ○ | ○ | ○ | ○ |
| PU2 Es ist mir wichtig, Gesundheitsunterlagen wie Arztbriefe und Befunde zentral speichern und teilen zu können. / It's important to me to be able to centrally store and share health records such as doctor's notes and medical records. | ○ | ○ | ○ | ○ |
| PU3 Die Nutzung der ePA spart mir Zeit bei der Verwaltung meiner Gesundheitsinformationen. / Using the ePA saves me time when managing my health information. | ○ | ○ | ○ | ○ |
| PU4 Durch die ePA konnte ich Doppeluntersuchungen vermeiden. / Thanks to the ePA, I was able to avoid duplicate tests. | ○ | ○ | ○ | ○ |
| PU5 Auch mit der ePA halte ich es für sinnvoll, Untersuchungen erneut durchführen zu lassen, selbst wenn frühere Ergebnisse verfügbar sind. / Even with the ePA, I think it makes sense to have examinations repeated, even if previous results are available. | ○ | ○ | ○ | ○ |

Zurück Weiter

Institute for Software Technology, University of Koblenz – 2025

Vorschau des Fragebogens "Fragebogen"

university of koblenz
Computer Science

38% ausgefüllt

**Szenario 2: Medikamentenübersicht dank E-Rezept**

Sie nehmen aktuell mehrere Medikamente ein, die von unterschiedlichen Ärzten verschrieben wurden. Früher mussten Sie sich die Namen und Dosierungen selbst merken oder auf einem Zettel notieren.

Mit der ePA sind all Ihre Medikamente automatisch in einer digitalen Übersicht gespeichert, die mit Ihrem E-Rezept verknüpft ist. Wenn Sie bei Ihrem Hausarzt eine neue Verschreibung erhalten, sieht dieser sofort, welche anderen Medikamente Sie aktuell einnehmen, und kann die Dosierung automatisch zur Vermeidung von Wechselwirkungen anpassen. Auch in der Apotheke können so Missverständnisse vermieden werden.

Scenario 2: Medication Overview Thanks to E-Prescriptions

You are currently taking several medications prescribed by different doctors. In the past, you had to memorize the names and dosages yourself or write them down on a piece of paper.

With the ePA, all your medications are automatically stored in a digital overview linked to your e-prescription. When you receive a new prescription from your primary care physician, they can immediately see which other medications you are currently taking and can automatically adjust the dosage to avoid interactions. This also helps prevent misunderstandings at the pharmacy.

| | Item | Stimme gar nicht zu | Stimme eher nicht zu | Stimme eher zu | Stimme voll und ganz zu | |
|---|---|---|---|---|---|---|
| PU6 | Die Nutzung der ePA erleichtert es mir, den Überblick über meine Medikamente zu behalten. | ○ | ○ | ○ | ○ | Using the ePA makes it easier for me to keep track of my medications. |
| PU7 | Ich finde es hilfreich, dass meine Medikamente automatisch in einer digitalen Übersicht gespeichert werden. | ○ | ○ | ○ | ○ | I find it helpful that my medications are automatically saved in a digital overview. |
| PR1 | Die ePA hilft mir, Missverständnisse bei Verschreibungen zu vermeiden und Wechselwirkungen zwischen Medikamenten zu erkennen. | ○ | ○ | ○ | ○ | The ePA helps me avoid misunderstandings regarding prescriptions and identify drug interactions. |
| BI1 | Ich beabsichtige, die ePA für das Management meiner Medikamente regelmäßig zu nutzen. | ○ | ○ | ○ | ○ | I plan to use the ePA regularly to manage my medications. |
| PR2 | Ich habe Angst, dass ich nicht steuern kann, welche Medikamente mein Arzt sehen kann. | ○ | ○ | ○ | ○ | I'm afraid that I can't control which medications my doctor can see. |

Zurück Weiter

Institute for Software Technology, University of Koblenz – 2025

Vorschau des Fragebogens "Fragebogen"

46% ausgefüllt

**Szenario 3: Behandlungen in Notfällen**

Sie sind im Urlaub in einer anderen Stadt und haben plötzlich starke Schmerzen. Da Sie sich bereits in der Vergangenheit wegen dieser Beschwerden in Behandlung befunden haben, wissen Sie, dass Informationen über frühere Untersuchungen und Diagnosen wichtig sein könnten.

Im Krankenhaus erklären Sie den Ärzten, dass diese Informationen in Ihrer ePA gespeichert sind. Der behandelnde Arzt kann sofort auf Ihre bisherigen Untersuchungen zugreifen und erkennt, welche Behandlungen und Medikamente bereits eingesetzt wurden. Dadurch spart das Krankenhaus wertvolle Zeit und kann direkt mit einer passenden Behandlung beginnen.

Scenario 3: Emergency Treatment

You are on vacation in another city and suddenly experience severe pain. Since you have been treated for these symptoms in the past, you know that information about previous examinations and diagnoses could be important.

At the hospital, you explain to the doctors that this information is stored in your ePA. The attending physician can immediately access your previous test results and see which treatments and medications have already been used. This saves the hospital valuable time and allows them to begin appropriate treatment right away.

| | Stimme gar nicht zu | Stimme eher nicht zu | Stimme eher zu | Stimme voll und ganz zu |
|---|---|---|---|---|
| PU8 Ich finde nützlich, dass Ärzte in Notfällen auf meine bisherigen Diagnosen und Behandlungen zugreifen können. | ○ | ○ | ○ | ○ |
| PU9 Die Nutzung der ePA reduziert das Risiko einer falschen Behandlung in Notfällen. | ○ | ○ | ○ | ○ |
| BI2 Ich würde die ePA auch in unerwarteten Situationen, wie einem Notfall nutzen. | ○ | ○ | ○ | ○ |

PU8: I find it helpful that doctors can access my previous diagnoses and treatments in emergencies.

PU9: Using the ePA reduces the risk of incorrect treatment in emergencies.

BI2: I would also use the ePA in unexpected situations, such as an emergency.

Zurück Weiter

Institute for Software Technology, University of Koblenz – 2025

university of koblenz
Computer Science

54% ausgefüllt

**Szenario 4: Datenschutz und individuelle Kontrolle**

Scenario 4: Data Protection and Individual Control

Ihre Gesundheitsdaten sind sensibel, und der Zugriff darauf sollte gezielt gesteuert werden. Über die ePA-App der Krankenkassen können individuelle Zugriffsrechte festgelegt werden. So kann beispielsweise der Hausarzt Zugriff auf die gesamte Krankengeschichte erhalten, während ein Facharzt nur ausgewählte Berichte einsehen kann.

Your health data is sensitive, and access to it should be carefully controlled. Individual access rights can be set via the health insurance companies' ePA app. For example, your primary care physician can be granted access to your entire medical history, while a specialist can only view selected reports.

Nutzung der ePA ohne die App:
Da Sie die ePA-App nicht nutzen möchten, haben Sie die Einstellungen gemeinsam mit einem Ansprechpartner Ihrer Krankenkasse vorgenommen. Sie behalten jederzeit die Kontrolle über Ihre Daten und können diese Rechte bei Bedarf anpassen.

Using the ePA without the app:
Since you do not wish to use the ePA app, you have configured the settings together with a representative from your health insurance provider. You retain control over your data at all times and can adjust these permissions as needed.

| | | Stimme gar nicht zu | Stimme eher nicht zu | Stimme eher zu | Stimme voll und ganz zu | |
|---|---|---|---|---|---|---|
| PR3 | Ich befürchte, dass ich nicht weiß, wie ich die Zugriffsrechte in der ePA verwalten kann. | ○ | ○ | ○ | ○ | I'm afraid I don't know how to manage access rights in the ePA. |
| PR4 | Ich fühle mich sicherer, wenn ich über die ePA-App individuell festlegen kann, wer welche Daten einsehen darf. | ○ | ○ | ○ | ○ | I feel more secure when I can use the ePA app to specify who is allowed to view which data. |
| AttentionCheckItem | Wählen Sie bitte die Antwortmöglichkeit „Stimme gar nicht zu“ aus. | ○ | ○ | ○ | ○ | Please select the response option “Strongly disagree.” |
| PR5 | Ich befürchte, die Kontrolle über meine Gesundheitsdaten zu verlieren. | ○ | ○ | ○ | ○ | I'm afraid of losing control over my health data. |
| S1 | Die Möglichkeit, die Einstellungen gemeinsam mit einem Ansprechpartner der Krankenkasse vorzunehmen, ist für mich wichtig. | ○ | ○ | ○ | ○ | The ability to set up the settings together with a representative from the health insurance company is important to me. |

Zurück Weiter

university of koblenz
Computer Science

62% ausgefüllt

**Szenario 5: Sicherheit der Gesundheitsdaten**

Bevor Sie die ePA genutzt haben, hatten Sie Bedenken hinsichtlich der sicheren Speicherung Ihrer Gesundheitsdaten. Sie erfahren, dass die Daten auf Servern in Deutschland nach hohen Sicherheitsstandards gespeichert und nur für klar definierte und legitimierte Zwecke genutzt werden.

Außerdem hören Sie, dass medizinische Unterlagen, die Sie früher selbst verwahren mussten, jetzt sicher in Ihrer ePA gespeichert sind.

Scenario 5: Security of Health Data

Before you started using the ePA, you had concerns about the secure storage of your health data. You learn that the data is stored on servers in Germany in accordance with high security standards and is used only for clearly defined and legitimate purposes.

You also learn that medical records, which you previously had to keep yourself,are now securely stored in your ePA.

| | | Stimme gar nicht zu | Stimme eher nicht zu | Stimme eher zu | Stimme voll und ganz zu | |
|---|---|---|---|---|---|---|
| PR6 | Ich habe Vertrauen in die Sicherheit und den Datenschutz meiner Gesundheitsdaten in der ePA. | ○ | ○ | ○ | ○ | I have confidence in the security and privacy of my health data in the ePA. |
| PR7 | Ich habe das Gefühl, dass meine Daten missbraucht werden könnten. | ○ | ○ | ○ | ○ | I have a feeling that my data might be misused. |
| PR8 | Die Speicherung nach höchsten Sicherheitsstandards erhöht mein Vertrauen und hilft, den Verlust wichtiger medizinischer Unterlagen zu vermeiden. | ○ | ○ | ○ | ○ | Storing data in accordance with the highest security standards increases my trust and helps prevent the loss of important medical records. |
| T1 | Ich habe kein Wissen darüber, welche Risiken bei der Nutzung der ePA auftreten können. | ○ | ○ | ○ | ○ | I have no knowledge of the risks that may arise from using the ePA. |
| T2 | Ich habe ausreichend Informationen über die Datenverarbeitung in der ePA erhalten. | ○ | ○ | ○ | ○ | I have received sufficient information about data processing in the ePA. |

Zurück Weiter

Institute for Software Technology, University of Koblenz – 2025

Vorschau des Fragebogens "Fragebogen"

university of koblenz
Computer Science

69% ausgefüllt

## Szenario 6: Nutzung der ePA in der Telemedizin

Scenario 6: Using the ePA in Telemedicine

Sie sind krank und vereinbaren eine Videosprechstunde mit einem Facharzt. Während der Konsultation fragt der Arzt, ob er Ihre ePA-Daten einsehen darf, um Vorbefunde und Laborwerte zu überprüfen. Der Arzt kann nur mit Ihrer Zustimmung auf die Daten zugreifen. Mit dieser Zustimmung kann dieser bei Bedarf neue Befunde in die ePA eintragen. Digitale Gesundheitsanwendungen (DiGA), die Sie nutzen, können ebenfalls nach Zustimmung der Daten in Ihrer ePA gespeichert werden.

You are sick and schedule a video consultation with a specialist. During the consultation, the doctor asks if they may view your ePA data to review previous findings and lab results. The doctor can only access the data with your consent. With this consent, the doctor can enter new findings into the ePA if necessary. Digital health applications (DiGA) that you use can also be stored in your ePA with your consent.

| | Item | Stimme gar nicht zu | Stimme eher nicht zu | Stimme eher zu | Stimme voll und ganz zu | English |
|---|---|---|---|---|---|---|
| PR9 | Ich halte es für sicher, dass Ärzte nur mit meiner Zustimmung auf die elektronische Patientenakte zugreifen können. | ○ | ○ | ○ | ○ | I am confident that doctors can only access the electronic health record with my consent. |
| BI3 | Ich kann mir vorstellen, die ePA für Videosprechstunde mit meinem Arzt zu nutzen. | ○ | ○ | ○ | ○ | I can imagine using the ePA for video consultations with my doctor. |
| PR10 | Ich finde es wichtig, dass ich selbst steuern kann, welche anderen digitalen Anwendungen Daten in meine ePA speichern können. | ○ | ○ | ○ | ○ | I think it’s important that I can control which other digital apps are allowed to save data to my ePA. |

Zurück Weiter

Institute for Software Technology, University of Koblenz – 2025

Vorschau des Fragebogens "Fragebogen"

university of koblenz
Computer Science

77% ausgefüllt

## Szenario 7: Soziale Einflüsse und Wahrgenommene Notwendigkeit

Sie haben in Gesprächen mit Freunden und Familienmitgliedern immer wieder gehört, wie hilfreich und praktisch es ist, eine elektronische Patientenakte zu haben. Diese haben alle in Ihren ePA-Datenbanken ihre Gesundheitsdaten gespeichert und können sie problemlos bei Ärzten und Kliniken vorzeigen.

Scenario 7: Social Influences and Perceived Necessity

In conversations with friends and family members, you have repeatedly heard how helpful and practical it is to have an electronic health record. They have all stored their health data in their ePA databases and can easily present it to doctors and clinics.

| | Stimme gar nicht zu | Stimme eher nicht zu | Stimme eher zu | Stimme voll und ganz zu | |
|---|---|---|---|---|---|
| PN2 Ich habe das Gefühl, dass die ePA einen echten Bedarf an der Speicherung meiner Gesundheitsdaten abdeckt. | ○ | ○ | ○ | ○ | I feel that the ePA meets a genuine need for the storage of my health data. |
| PU10 Je mehr ich über die ePA erfahre, desto nützlicher erscheint mir ihre Nutzung für meine Gesundheitsversorgung. | ○ | ○ | ○ | ○ | The more I learn about the ePA, the more useful it seems to me for my healthcare. |
| SI1 Der Einfluss von Ärzten, Familie und Freunden hat meine Entscheidung beeinflusst, die ePA zu nutzen. | ○ | ○ | ○ | ○ | The influence of doctors, family, and friends played a role in my decision to use the ePA. |
| PU11 Die Nutzung der ePA verbessert meine Erfahrung im Gesundheitswesen insgesamt. | ○ | ○ | ○ | ○ | Using the ePA improves my overall experience with the healthcare system. |

Zurück Weiter

Vorschau des Fragebogens "Fragebogen"

university of koblenz
Computer Science

85% ausgefüllt

Scenario 8: Use of ePA Data for Medical Research

**Szenario 8: Nutzung der ePA-Daten für die medizinische Forschung**

Bei der Einrichtung Ihrer ePA erhalten Sie die Information, dass Ihre Gesundheitsdaten standardmäßig für Forschungszwecke pseudonymisiert weitergeleitet werden. Dabei werden Ihre persönlichen Daten durch eine Identifikationsnummer ersetzt. Die Weiterleitung dieser Daten ist standardmäßig aktiviert, aber das können Sie jederzeit ändern. Bei der Einrichtung der ePA werden Sie über diese Funktion informiert.

Diese Informationen regen Sie zum Nachdenken an. Einerseits könnte Ihre Zustimmung die Forschung medizinischer Innovation vorantreiben, andererseits machen Sie sich Gedanken über den Datenschutz und die Kontrolle Ihrer persönlichen Daten.

When setting up your ePA, you are informed that your health data will be shared for research purposes in a pseudonymized form by default. In this process, your personal data is replaced with an identification number. The sharing of this data is enabled by default, but you can change this at any time. You are informed about this feature when setting up the ePA.

This information gives you pause for thought. On the one hand, your consent could advance medical innovation research; on the other hand, you have concerns about data protection and control over your personal data.

| Code | Item | Stimme gar nicht zu | Stimme eher nicht zu | Stimme eher zu | Stimme voll und ganz zu | English |
|---|---|---|---|---|---|---|
| BI4 | Ich bin bereit, meine pseudonymisierten ePA-Daten für medizinische Forschungszwecke freizugeben. | ○ | ○ | ○ | ○ | I am willing to share my pseudonymized ePA data for medical research purposes. |
| PR11 | Ich habe Bedenken, dass meine Gesundheitsdaten trotz Pseudonymisierung missbraucht werden könnten. | ○ | ○ | ○ | ○ | I am concerned that my health data could be misused despite pseudonymization. |
| BI5 | Die Möglichkeit, dass meine Daten zur Entwicklung neuer Therapie beitragen, motiviert mich, meine Daten freizugeben. | ○ | ○ | ○ | ○ | The possibility that my data could contribute to the development of new treatments motivates me to share my data. |
| PR12 | Ich habe kein Vertrauen in die Sicherheit und den Datenschutz meiner Gesundheitsdaten in der ePA. | ○ | ○ | ○ | ○ | I have no confidence in the security and privacy of my health data in the ePA. |
| T3 | Mir ist es wichtig, selbst zu entscheiden, ob meine ePA-Daten für die Forschung genutzt werden dürfen. | ○ | ○ | ○ | ○ | It is important to me to decide for myself whether my ePA data may be used for research. |
| T4 | Ich vertraue darauf, dass meine Daten nur zu wissenschaftlichen Zwecken genutzt werden. | ○ | ○ | ○ | ○ | I trust that my data will only be used for scientific purposes. |
| T5 | Es ist mir wichtig, jedes Mal eine Benachrichtigung zu erhalten, wenn meine Daten für Forschungszwecke genutzt werden. | ○ | ○ | ○ | ○ | It is important to me that I receive a notification every time my data is used for research purposes. |

Zurück Weiter

Institute for Software Technology, University of Koblenz – 2025

Vorschau des Fragebogens "Fragebogen"

university of koblenz
Computer Science

92% ausgefüllt

## Zusätzliche Fragen zu Erfahrungen und Einstellungen

Additional questions on experiences and attitudes

| | Stimme gar nicht zu | Stimme eher nicht zu | Stimme eher zu | Stimme voll und ganz zu | |
|---|---|---|---|---|---|
| PEOU1 Die Nutzung der ePA ist für mich einfach und unkompliziert. | ○ | ○ | ○ | ○ | Using the ePA is easy and straightforward for me. |
| PEOU2 Ich finde es leicht, die Funktionen der ePA zu verstehen. | ○ | ○ | ○ | ○ | I find it easy to understand the features of the ePA. |

Welche Vorteile der ePA motivieren Sie am meisten dazu, die ePA zu nutzen?

OpenQuestionAdvantages

Which benefits of the ePA motivate you the most to use the ePA?

Haben Sie Bedenken oder Hindernisse in Bezug auf die Nutzung der ePA? Wenn ja, welche?

OpenQuestionConcerns

Do you have any concerns or obstacles regarding the use of the ePA? If so, what are they?

Zurück Weiter

Institute for Software Technology, University of Koblenz – 2025

Vorschau des Fragebogens "Fragebogen"

# Vielen Dank für Ihre Teilnahme!

Wir möchten uns ganz herzlich für Ihre Mithilfe bedanken.

Ihre Antworten wurden gespeichert, Sie können das Browser-Fenster nun schließen.

Thank you very much for participating!

We would like to sincerely thank you for your help.

Your responses have been saved; you can now close the browser window.

Institute for Software Technology, University of Koblenz – 2025
