# Supplementary material for: Health Dataspace Technology Acceptance Model (HDS-TAM): Development and Case Evaluation Study of Patient Acceptance of Germany’s Electronic Patient Record
Source: JMIR Med Inform. 2026 Nov 2;14:e99366. doi: 10.2196/99366 (PMC13726799; doi:10.2196/99366)
Supplement: Supplementary file 4 [file medinform-v14-e99366-s004.pdf]

HDS-TAM ePA Case Evaluation Survey: Detailed Sample Characteristics

Status	Gender	Count	%
Valid	female	89	59.7%
	male	50	33.6%
	diverse	1	0.7%
	Total	140	94.0%
Missing	no answer	9	6.0%
**Status**	**Age (Years)**	**Count**	**%**
Valid	<=19	6	4.0%
	20-24	54	36.2%
	25-29	41	27.5%
	30-34	12	8.1%
	35-39	6	4.0%
	40-44	7	4.7%
	45-49	3	2.0%
	50-54	2	1.3%
	55-59	5	3.4%
	60-64	6	4.0%
	>=65	1	0.7%
	Total	143	96.0%
Missing	no answer	6	4.0%
**Status**	**Occupation**	**Count**	**%**
Valid	School Student	1	0.7%
	Apprentice	5	3.4%
	University Student	80	53.7%
	Employee	51	34.2%
	Civil Servant	3	2.0%
	Self-Employed	1	0.7%
	Unemployed	2	1.3%
	Other	5	3.4%
	Total	148	99.3%
Missing	no answer	1	0.7%
**Status**	**ePA Usage**	**Count**	**%**
Valid	yes	35	23.5%
	no	113	75.8%
	Total	148	99.3%
Missing	no answer	1	0.7%
**Status**	**Previous Illness**	**Count**	**%**
Valid	yes	41	27.5%
	no	108	72.5%
	Total	149	100.0%
Missing	no answer	0	0.0%
**Status**	**Regular Medication**	**Count**	**%**
Valid	yes	50	33.6%
	no	99	66.4%
	Total	149	100.0%
Missing	no answer	0	0.0%
**Status**	**Technical Knowledge**	**Count**	**%**
Valid	very high	15	10.1%
	high	50	33.6%
	medium	75	50.3%
	low	6	4.0%
	very low	3	2.0%
	Total	149	100.0%
Missing	no answer	0	0.0%
**Sample Size**		**149**	**100.0%**
