# Supplementary material for: Health Dataspace Technology Acceptance Model (HDS-TAM): Development and Case Evaluation Study of Patient Acceptance of Germany’s Electronic Patient Record
Source: JMIR Med Inform. 2026 Nov 2;14:e99366. doi: 10.2196/99366 (PMC13726799; doi:10.2196/99366)
Supplement: Supplementary file 5 [file medinform-v14-e99366-s005.pdf]

HDS-TAM ePA Case Evaluation Survey: Missing Data Details																																														
**# of cases**	**PN1**	**PU8**	**PU9**	**Support**	**TechUnderstanding**	**PR4i**	**Medication**	**HealthIssues**	**PU_score**	**BI2**	**PR3**	**PR5**	**EPAUse**	**PU2**	**PR7**	**PU7**	**PR6i**	**T1i**	**PU6**	**PR2**	**PR8i**	**T2**	**BI1**	**PU3**	**PR10**	**PR9i**	**PR1i**	**PU1**	**BI3**	**PU10**	**AgeGroup**	**PU5**	**SI1**	**T3**	**T5**	**PN2**	**BI4**	**PR11**	**BI5**	**PR12**	**T4**	**PU4**	**PU11**	**Gender**	**PEOU1**	**PEOU2**	**# of missing values per case**
111	-	-	-	-	-	-	-	-	-	-	-	-	-	-	-	-	-	-	-	-	-	-	-	-	-	-	-	-	-	-	-	-	-	-	-	-	-	-	-	-	-	-	-	-	-	-	**0**
6	-	-	-	-	-	-	-	-	-	-	-	-	-	-	-	-	-	-	-	-	-	-	-	-	-	-	-	-	-	-	-	-	-	-	-	-	-	-	-	-	-	-	-	-	X	X	**2**
3	-	-	-	-	-	-	-	-	-	-	-	-	-	-	-	-	-	-	-	-	-	-	-	-	-	-	-	-	-	-	-	-	-	-	-	-	-	-	-	-	-	-	-	X	-	-	**1**
1	-	-	-	-	-	-	-	-	-	-	-	-	-	-	-	-	-	-	-	-	-	-	-	-	-	-	-	-	-	-	-	-	-	-	-	-	-	-	-	-	-	X	-	-	-	-	**1**
1	-	-	-	-	-	-	-	-	-	-	-	-	-	-	-	-	-	-	-	-	-	-	-	-	-	-	-	-	-	-	-	-	-	-	-	X	-	-	-	-	-	-	-	-	-	-	**1**
1	-	-	-	-	-	-	-	-	-	-	-	-	-	-	-	-	-	-	-	-	-	-	-	-	-	-	-	-	-	-	-	-	-	X	X	-	X	X	X	X	X	-	-	-	X	X	**9**
1	-	-	-	-	-	-	-	-	-	-	-	-	-	-	-	-	-	-	-	-	-	-	-	-	-	-	-	-	-	-	-	X	-	-	-	-	-	-	-	-	-	-	-	-	-	-	**1**
1	-	-	-	-	-	-	-	-	-	-	-	-	-	-	-	-	-	-	-	-	-	-	-	-	-	-	-	-	-	-	-	X	-	-	-	-	-	-	-	-	-	-	-	-	X	X	**3**
1	-	-	-	-	-	-	-	-	-	-	-	-	-	-	-	-	-	-	-	-	-	-	-	-	-	-	-	-	-	-	-	X	-	-	-	-	-	-	-	-	-	X	-	-	-	-	**2**
6	-	-	-	-	-	-	-	-	-	-	-	-	-	-	-	-	-	-	-	-	-	-	-	-	-	-	-	-	-	-	X	-	-	-	-	-	-	-	-	-	-	-	-	X	-	-	**2**
1	-	-	-	-	-	-	-	-	-	-	-	-	-	-	-	-	-	-	-	-	-	-	-	-	-	-	X	-	-	-	-	-	-	-	-	-	-	-	-	-	-	-	-	-	-	-	**1**
1	-	-	-	-	-	-	-	-	-	-	-	-	-	-	-	-	-	-	-	-	-	-	-	-	-	-	X	-	-	X	-	-	X	X	X	X	X	X	X	X	X	-	X	-	X	X	**14**
3	-	-	-	-	-	-	-	-	-	-	-	-	-	-	-	-	-	-	-	-	-	-	-	-	X	X	-	-	X	X	-	-	X	X	X	X	X	X	X	X	X	-	X	-	X	X	**16**
1	-	-	-	-	-	-	-	-	-	-	-	-	-	-	-	-	-	-	-	-	-	-	-	X	-	-	-	X	-	-	-	X	-	-	-	-	-	-	-	-	-	X	-	-	X	X	**6**
1	-	-	-	-	-	-	-	-	-	-	-	-	-	-	-	-	-	-	-	-	-	-	X	-	-	-	X	-	-	-	-	-	-	-	-	-	-	-	-	-	-	X	-	-	-	-	**3**
1	-	-	-	-	-	-	-	-	-	-	-	-	-	-	-	-	-	-	-	-	-	-	X	X	-	-	-	X	-	-	-	-	-	-	-	-	-	-	-	-	-	X	X	-	X	X	**7**
1	-	-	-	-	-	-	-	-	-	-	-	-	-	-	-	-	-	-	-	-	-	X	-	-	-	-	-	-	-	-	-	-	-	-	-	-	-	-	-	-	-	-	-	-	X	X	**3**
1	-	-	-	-	-	-	-	-	-	-	-	-	-	-	-	-	-	-	X	-	-	-	-	-	-	-	-	X	-	-	-	-	-	-	-	-	-	-	-	-	-	X	-	-	-	-	**3**
1	-	-	-	-	-	-	-	-	-	-	-	-	-	-	X	-	X	X	-	-	X	X	-	-	X	X	-	-	X	X	-	-	X	X	X	X	X	X	X	X	X	-	X	-	X	X	**21**
1	-	-	-	-	-	-	-	-	-	-	-	-	-	-	X	X	X	X	X	X	X	X	X	X	X	X	X	X	X	X	-	X	X	X	X	X	X	X	X	X	X	X	X	-	X	X	**30**
1	-	-	-	-	-	-	-	-	-	-	-	-	-	X	-	-	-	-	-	-	-	-	-	-	-	-	-	-	-	-	-	-	-	-	-	-	-	-	-	-	-	-	-	-	-	-	**1**
1	-	-	-	-	-	-	-	-	-	-	-	-	X	-	-	-	-	-	-	-	-	-	-	-	-	-	-	-	-	-	-	-	-	-	-	-	-	-	-	-	-	-	-	-	-	-	**1**
1	-	-	-	-	-	-	-	-	-	-	-	X	-	-	-	-	-	-	-	-	-	-	-	-	-	-	-	-	-	-	-	-	X	-	-	-	-	-	-	-	-	-	X	-	X	X	**5**
1	-	-	-	-	-	-	-	-	-	-	X	-	-	-	-	-	-	-	-	X	-	X	-	X	-	-	-	X	-	-	-	X	-	-	-	X	X	X	X	X	X	X	X	-	X	X	**16**
1	-	-	-	-	-	-	-	-	-	X	-	-	-	X	-	X	-	-	X	X	X	-	X	X	-	-	X	X	X	-	-	X	-	-	-	-	-	-	-	-	-	X	-	-	-	-	**13**
**# of missing values per item**	**0**	**0**	**0**	**0**	**0**	**0**	**0**	**0**	**0**	**1**	**1**	**1**	**1**	**2**	**2**	**2**	**2**	**2**	**3**	**3**	**3**	**4**	**4**	**5**	**5**	**5**	**5**	**6**	**6**	**6**	**6**	**7**	**7**	**7**	**7**	**8**	**8**	**8**	**8**	**8**	**8**	**9**	**9**	**9**	**19**	**19**	**216**
	Legend:		X	item value missing																																										
			-	item value not missing																																										
