# Supplementary material for: Health Dataspace Technology Acceptance Model (HDS-TAM): Development and Case Evaluation Study of Patient Acceptance of Germany’s Electronic Patient Record
Source: JMIR Med Inform. 2026 Nov 2;14:e99366. doi: 10.2196/99366 (PMC13726799; doi:10.2196/99366)
Supplement: Supplementary file 6 [file medinform-v14-e99366-s006.pdf]

HDS-TAM ePA Case Evaluation: Answers to Open Questions

(Original German answers, translation to English starts on page 5)

Welche Vorteile der ePA motivieren Sie am meisten dazu, die ePA zu nutzen?

- Insgesamt finde ich ePA sehr nützlich, weil man auf Überblick über seine Gesundheitlichen Daten.
  Anhand Daten von der ePA könnte in Zukunft viele neue Diagnose entstehen. Das wird sicherlich Menschen helfen.
- Die Daten an einem Ort zu speichern und jederzeit abrufbar zu haben.
- Nutzung zur Medikamenten Verschreibung bei einen Arzt Wechsel.
- Es ist praktisch, dass man selbst keine Ahnung haben muss und die Daten elektronisch übermittelt werden können. Vor allem bei Vorbehandlungen, die länger zurückliegen und man ggf. selbst nicht mehr weiß, was da gemacht wurde, ist die ePA bestimmt sehr hilfreich. Weil manche Ärzte schwer lesbare Handschriften haben, ist es außerdem angenehmer, alles digital geschrieben lesen zu können.
- Weil alles an einem Ort gespeichert ist und man keine Zettelwirtschaft organisieren muss.
  Außerdem kann dies für die Behandlung erleichternd sein.
- Ich nutze die ePA nicht aktiv da ich keine Ahnung habe.
- Ich hätte die ePA derzeit noch nicht nutzen, da ich privat versichert bin, obwohl es mir sehr helfen würde, da ich derzeit bei vielen verschiedenen Ärzten in Behandlung bin und die Kommunikation der Ärzte sehr verbessern würde und auch ein besseres Verständnis vorhandener wäre.
- Leichter Zugriff auf gesamte Gesundheitshistorie Überblick über Dokumente und Befunde, kein Blätterchaos
- Übersichtliche Speicherung von gesundheitsrelevanten Daten
- Zentrale Speicherung aller relevanten Befunde und genereller Gesundheitsdaten potenzielle Weiterentwicklung von Therapien
- Einfacher Zugriff, Schnellere Ergebnisse, Alles Einsehbar
- Alle notwendigen Daten können in Notsituationen schnell abgerufen werden, um dem Patienten schnellstmöglich zu helfen und keine Zeit zu verschwenden. Wenn man bei unterschiedlichen Ärzten ist, dann wäre es vielleicht sogar von Vorteil bei der Behandlung, wenn diese die ganzen Akte sehen, weil einige Symptome Ausdruck von einer anderen Krankheit sein können. So hätte der Arzt also einen ganzheitlichen Blick auf den Patienten und könnte eventuell so besser feststellen, wann eine Überweisung an einen anderen Arzt notwendig ist bzw. kann dieser die Behandlung so durchführen, dass dem Patienten besser geholfen wird und andere Ursachen evtl. mit einbezogen werden können, die der Patient als Laie, dem Arzt so nicht mitgeteilt hat. Ich weiß aus meiner Erfahrung mit dem E-Rezept, dass die versprochene Schnelligkeit und der Abbau der Bürokratie nicht funktioniert. Ich muss darauf warten, dass das Rezept erst durch meinen Arzt freigeschaltet wird, damit nicht das
- Kontrolle über E-Rezepte und Medikamente und die Übersicht der Medikamente, sodass man keine Wechselwirkungen von Medikamenten erleben muss.
- Ich nutze es bisher nicht.
- Übersicht, Aufbewahrung, Langzeit.
- Möglichkeit Befunde mit verschiedenen Ärzten zu teilen. Medikamentenübersicht für verschiedene Ärzte.
- Alles auf einen Blick zu haben.
- Erleichterung im Alltag.
- Ordnung der Daten und der digitale Zugriff
- Ärzte sehen direkt die Infos. Nicht durch mich als "Stille Post". Alles ist an einem Ort gespeichert.
- Sammeln alle Gesundheitsinformationen und Medikation sowie die Einsichtnahme durch andere Ärzte vor allem in Notfällen, z.B. bei einem Unfall oder im Urlaub.
- Das ich volle Einsicht in meine Unterlagen habe.
- Ich denke es ist einfach zeitgemäß und alternativlos.
- Intersektionalität des Gesundheitswesen wird verstärkt. Insb. für Medikamenten und Notfallmanagement über alle Maße sinnvoll!!! (oder wenn Person kognitiv eingeschränkt sind)
- Die zentrale Speicherung aller medizinischen Daten vereinfacht das aufbewahren der Arztberichte.
- Vereinfachte Datenablage, Digitalisierung der Prozessekeine
- Wie zu Beginn angegeben nutze ich die ePA aktuell noch nicht.
- Keine, da die nachgewiesen unsichere Implementierung (siehe Vorträge des CCC zum Thema) überwiegen. Ich habe Alter oder eine Behinderung überwiegen vermutlich die Vorteile. Solange ich für mich selbst entscheiden kann, kann ich auch selbst auf meine Daten aufpassen.
- Man hat alles auf einen Blick und kann sich jederzeit Details vergangener Behandlungen, Krankmeldungen, Medikationen etc. in Ruhe ansehen. Somit habe ich die Möglichkeit, mich selbst besser zu informieren kann und auch besser verstehen kann, was ein Ärztin dokumentiert hat. Besonders in Fällen, in denen man sich nicht gut genug aufgeklärt fühlt oder sich nicht ernstgenommen fühlt, ist es hilfreich alles schriftliche danach einsehen zu können. So fühlt man sich sicherer.
- Die Einsicht auf meine Medikamente

- Alle Untersuchungs- und Behandlungsunterlagen an einem Ort. Vergangene Behandlungen können vom Arzt leichter eingesehen werden, dass sich alles sehen kann.
- Selbst den Überblick haben, weniger Zettelwirtschaft zu Hause
- Es ist einfach und effizient war es auch sinnvoller als Zeit der Digitalisierung.
- Der einfache Zugriff auf alle Akten in Notfällen. Der Überblick über alle eingenommenen Medikamente und mögliche Wechselwirkungen. Dass keine wichtigen Informationen verloren gehen.
- Das es Digital ist
- Übersicht über meine Krankengeschichte; keine Anfragen bei Ärzt*innen mit Bitte um Weitergabe meiner Daten
- Die Zusammenfassung der medizinischen Angelegenheiten ist in großer Vorteil für viele Menschen. Auch für Ärzte ist es hilfreich mehr Informationen über die Patienten zu erhalten.
- Einsicht in Diagnosen, Gesundheit auf einem Blick
- Zugriff auf Daten im Notfall (bewusst). Automatische, unkomplizierte und vollständige Weitergabe von Daten
- Übersicht über Krankenhistorie, Einblick in Diagnosen und Therapien
- Übersicht über Medikamente, Diagnosen, Befunde an einem Ort. Weiterleitung der Gesundheitsdaten für Forschungszwecke. Einfache Einsicht für Arztpersonal auf momentan eingenommene Medikamente, Vorerkrankungen, frühere Beschwerden, sodass eine besser angepasste Behandlung erfolgen kann.
- Alle meine gesundheitlichen Informationen sind gebündelt an einem Ort und können jederzeit eingesehen, aktualisiert und verwaltet werden, ohne dass etwas verloren geht.
- Zentrale Dokumentenablage. Erinnerungen an Impfungen, Gesundheitsvorsorge etc. Vermeidung von Doppel- oder Fehlbehandlungen
- Alle medizinischen Dokumente zentral an einem Ort, immer verfügbar und leicht mit entsprechenden Personen teilbar.
- Zentraler Zugriff
- Organisation & zentrale Verwaltung aller Gesundheitsinformationen – selektives Teilen der Daten
- Alle Daten an einem Ort zu haben, und immer abrufen zu können.
- Ich habe bei Notfällen alles im Griff
- Bessere und einfache Kommunikation zwischen Ärzten
- Dass keine Daten verloren gehen können.

Haben Sie Bedenken oder Hemmnisse in Bezug auf die Nutzung der ePA? Wenn ja, welche?

- Die Einstellung ist ein bisschen zeitaufwendig und braucht ein bisschen technische Kenntnisse.
- Ich denke, Ärzte müssen sehr gut unterrichtet werden, wie der Umgang mit der ePA funktioniert, damit das alles so gut funktioniert, wie man sich das vorstellt. Bei Menschen, die eventuell nicht so gut mit dieser Technik umgehen, führt das wahrscheinlich nur zu mehr Problemen.
- Da mein Hausarzt noch nicht einmal die Daten ansieht, die ich selber bei vorherigen Besuchen eingetragen habe, und mir häufig die gleichen Fragen mehrmals stellt, wage ich zu bezweifeln, dass in diese Akte genau reinschauen wird.
- Kenne die ePA nur von Bezeichnung her, mehr weiß ich darüber nicht.
- Eigentlich nicht. Ich denke nur, dass meine Hausarztpraxis große Probleme haben wird, da diese Praxis derzeit nicht mal in der Lage ist, Emails zu erhalten und technisch sehr rückständig ist.
- Eine ePA kann dazu führen, dass ärztliches Personal sich nur auf die dort verfassten und Differenzialdiagnosen außer Acht lassen kann. Gerade in der Inneren Medizin und Psychiatrie gibt es Symptome, die für mehrere Erkrankungen stehen. Außerdem bin ich mir auch nicht sicher, ob Einträge zu Erkrankungen und Medikamenten ebenfalls die Namen der behandelnden Person stehen. Es sollte transparent sein, wer was warum eingetragen und gelöscht hat.
- Die App meiner Krankenkasse stürzt oft ab oder ich kann sie gar nicht öffnen.
- Datenlecks und Missbrauch meiner Daten. Bei Zusatzversicherungen wie Arbeitsunfähigkeit.
- Ja, Verkauf meiner Daten, allgemeine Weitergabe der Daten.
- Ich frage mich, wie man gewährleisten will, dass kein unrechtmäßiger Zugriff auf die Daten der Patienten stattfindet. Alles, was elektronisch abgespeichert wird, ist meiner Meinung nach immer der Gefahr eines Angriffs von außen ausgesetzt, auch wenn man die notwendigen Sicherheitsmaßnahmen angreift, um diese Daten zu schützen. Die Gefahr, so klein sie auch ist, besteht also immer. Im Falle eines solchen unberechtigten Zugriffs könnten diese hochsensiblen Daten für Zwecke verwendet werden, die nicht im Interesse des Patienten sind oder schlimmstenfalls sogar gegen dieses verwendet werden. Wenn also beispielsweise meine Daten in die Hände einer Bank fallen würden, die mir dann aufgrund meines gesundheitlichen Zustands die Kreditwürdigkeit abspricht, wäre das wohl ein nicht allzu unwahrscheinliches Szenario, womit wahrscheinlich sehr viele Patienten unzufrieden wären. Ich frage mich außerdem, wie diese gesundheitlichen Daten überhaupt Personen anonymisiert werden und ob es nicht doch in irgendeiner Form Manipulationen gibt.
- Ja, ich habe Bedenken, da ich die ePA sehr kritisch nutze und sensibler Daten habe und bin nicht vollständig davon überzeugt, dass wirklich alle wichtigen Daten der Hacker-Angriffe wirklich geschützt werden. Gesundheitsdaten gehen niemanden an außer mich und meinen behandelnden Arzt, was an meiner Skepsis bzgl. der Datensicherheit der App liegt.
- Ich habe keine Bedenken. Da ich seit Jahren nur zu Routineuntersuchungen zum Arzt gehe und auch keine Medikamente nehme, besteht aktuell kein Bedarf.
- Datenschutz bzgl. Sicherheit der Infrastruktur.
- Datenschutz und Sicherheit.
- Das die Daten in falsche Hände kommen, oder ich diese schwer bis unübersichtlich für mich erscheinen.
- Missbrauch durch Dritte
- Negative Auswirkungen auf meine Versicherung / Leistungen durch gespeicherte Infos.
- Das der Datenzugang begrenzt ist und nur von mir freigegebene Personen auf mir freigegebene Daten zugreifen können.
- Das Vertrauen in die Nutzung der ePA höht natürlich stark mit dem Vertrauen in das gesamte System. So können beispielsweise politische Änderungen dazu führen, dass auch das Misstrauen in einen verantwortungsvollen Umgang der Daten führt.
- Befürchtung, dass Daten nicht (nur) für gemeinnützige Forschungs-, sondern auch bzw. primär für Markt-Meinungsforschung oder kommerziell missbraucht werden. Von hybrider Kriegsführung sind auch Server in Deutschland nicht ausgenommen.
- Unausgereiftes System, Bedenken hinsichtlich Datenschutz und Sicherheit.
- Aufgrund der Tatsache, dass in vielen anderen Bereichen online Lösungen häufig Störungen haben denke ich, dass eine gänzliche Umstellung nur mit der Zeit umgesetzt werden kann. Denn bei einem Serverabsturz könnten durchaus Daten gelöscht werden, die für den einzelnen Patienten wichtig sein könnten.
- Cyberangriffe
- Keine Ende-zu-Ende-Verschlüsselung, diverse gravierende Sicherheitsprobleme (auch aus Sicht der Systemarchitektur), konsequentes Herunterspielen seitens der Verantwortlichen, Datenschutz wird unter dem Motto „Mit KI wird alles toll" ohne das ein wirkliches Verständnis der Thematik sichtbar wird, pseudonyme Sicherung der Daten ist nicht ausreichend – Anonymisierung wäre erforderlich, siehe Chat-Transcripts, ich gehe davon aus, dass diese Daten nicht nur für Therapien, sondern auch für andere Zwecke verwendet werden sollen (siehe Gespräche mit Microsoft und Google), opt-out statt opt-in, zukünftige potentielle Benachteiligung von Personen ohne ePA, gesellschaftliche Stigmatisierung von Personen ohne ePA.
- Fehlende Kompetenz der Gematik bezüglich Sicherheit. Eine APP macht nichts automatisch besser als eine Webseite

- Ja, Bedenken bzgl. Datenlecks durch Schwachstellen in der technischen Umsetzung. Außerdem Bedenken ob bekannt gewordene Probleme zeitnah wahrgenommen und behoben werden (siehe https://www.ccc.de/en/updates/2024/ende-der-epa-experimente). Schließlich habe ich Bedenken, dass der Zugang zur ePA in Arztpraxen flächendeckend und zeitnah umgesetzt wird.
- Ich möchte keine zentrale Speicherung sensibler Daten. Ich würde eine lokale verschlüsselte Speicherung auf einem eigenen Gerät oder Karte bevorzugen. Außerdem habe ich Bedenken, dass in der Zukunft nicht alle Daten so verwaltet werden können wie ich es möchte, wenn z. B. Krankenkassen davon einen Nutzen hätten.
- Grundsätzlich stehe ich dem positiv gegenüber. Aber es gibt immer ein Risiko, dass Daten missbraucht werden. Sicherheitsmaßnahmen können nie hundertprozentigen Schutz gewährleisten. Insofern habe ich leichte Bedenken, was passieren könnte, falls sich jemand an den Daten bemächtigt, der/die keinen Zugang haben sollte.
- Kein System ist zu 100 % sicher. Server mit sensiblen medizinischen Daten können ein lukratives Angriffsziel bieten und wären unter demgemäß potenziell eher gefährdet, angegriffen zu werden. Zudem, was passiert bei Ausfall der Server? Wäre mein Arzt in diesem Moment „blind“?
- Die ePA speichert personenbezogene Daten außerhalb meiner Kontrolle.
  Die Daten sind leichter zugänglich für Dritte durch die Zentralisierung.
  Die Verwaltung der Zugriffsrechte ist nicht selektiv genug implementiert.
  Es sind meiner größeren Sicherheitslücken bekannt und offen.
- Ja man kann heute niemandem mehr trauen
- Datenschutz, Sicherheit der gespeicherten Daten gegenüber Dritten
- App sehr umständlich, was teilweise auf (sinnvolle) Sicherheitsvorkehrungen zurückzuführen ist – generell war der Einrichtungs-- und Identifizierungsprozess sehr aufwendig. Außerdem fraglich, ob es durch die ePA nicht zu erhöhtem Aufwand für Arztpraxen kommt – Zeit, die dann für Patienten fehlt.
- Bezüglich der Daten ist ja auch teils gängig, dass Daten an andere Länder oder im Schwarzmarkt verkauft werden
- Ich zweifle an der Sicherheit der ePA. Die personenbezogenen Daten extrem vieler Menschen auf einem Haufen können nach einem Ziel für Cyber-Kriminalität.
- Langfristig könnten die Daten theoretisch dafür genutzt werden, mich als Risikopatient o. ä. zu identifizieren und andere Beiträge zu berechnen; Missbräuchliche Weitergabe; Datenlecks
- Ich habe Sorge, dass aufgrund von Diagnosen, die nicht immer richtig sein müssen, vielleicht falsche Schlüsse von Ärzten und Gesundheitsmitarbeitern gezogen werden könnten.
- Leaks Hacker
- Die Sicherheit der Daten ist noch nicht gewährleistet (wodurch sich die Einführung auch verzögert)
  Eine zentrale Speicherung ist immer mit Risiken für Datenmissbrauch verbunden
- Technologie ist auch nicht fehlerfrei. Datenleaks, Kurzschlüsse bei denen Daten nicht gespeichert werden, unbedingt ggf. WLAN-Verbindung.
- Aufwendiger Registrierungsprozess; wenig Aufklärung über Datenschutz
- Solange alles nach neusten Sicherheitsstandards entwickelt ist und durch ausreichende Backup-Systeme gesichert ist, habe ich keine Bedenken.
- Fehlende Transparenz – Zu hohe Unsicherheiten
- Nein, ich finde es ist an der Zeit für eine solche Technologie, und keine Technologie ist zum Release perfekt.
- Meine Bedenken sind, dass es evtl. daran scheitern wird, alle Akteure der ePA ausreichend zu schulen bzw. dass durch unsere „never-change-a-running-system“-Mentalität für eine gewisse Zeit eine Doppelbelastung entsteht. Gut wäre es, wenn man die Zeitspanne der Umgewöhnung durch möglichst klein hält, sodass möglichst wenig Chaos entsteht

HDS-TAM ePA Case Evaluation: Answers to Open Questions

(Automatically translated to English by Microsoft CoPilot and checked by the authors)

Which benefits of the ePA motivate you most to use the ePA?

- Overall, I find the ePA very useful because it gives an overview of one's health-related data.
  Based on data from the ePA, many new diagnoses could emerge in the future. This will certainly help people.
- Storing the data in one place and being able to access it at any time.
- Use for prescribing medication when changing doctors.
- It is practical that you do not have to know everything yourself and that the data can be transmitted electronically. Especially in the case of prior treatments that took place a long time ago and that you yourself may no longer remember, the ePA is certainly very helpful. Since some doctors have hard-to-read handwriting, it is also more pleasant to be able to read everything digitally.
- Because everything is stored in one place and you do not have to organize paperwork.
  This can also make treatment easier.
- I do not actively use the ePA because I have no idea about it.
- I would not yet use the ePA at this time because I am privately insured, although it would help me a lot, since I am currently being treated by many different doctors and communication between doctors would be greatly improved and understanding would also be better.
- Easy access to the complete medical history, overview of documents and findings, no paper chaos.
- Clear storage of health-relevant data.
- Central storage of all relevant findings and general health data, potential further development of therapies.
- Easy access, faster results, everything viewable.
- All necessary data can be retrieved quickly in emergency situations in order to help the patient as quickly as possible and not waste time. If you see different doctors, it could even be an advantage for treatment if they could see the entire record, because some symptoms can be an expression of another illness. This would give the doctor a holistic view of the patient and potentially allow them to better determine when a referral to another doctor is necessary or to carry out treatment in a way that better helps the patient and possibly includes other causes that the patient, as a layperson, did not communicate to the doctor. From my experience with e-prescriptions, I know that the promised speed and reduction of bureaucracy does not work. I have to wait for the prescription to first be activated by my doctor so that it does not [maximum length of answer reached]
- Control over e-prescriptions and medications and an overview of medications so that you do not experience interactions between medications.
- I have not used it so far.
- Overview, storage, long-term.
- Possibility to share findings with different doctors. Medication overview for different doctors.
- Having everything at a glance.
- Easier daily life.
- Organization of data and digital access.
- Doctors see the information directly. Not through me as "Chinese whispers." Everything is stored in one place.
- Collecting all health information and medication as well as access by other doctors, especially in emergencies, e.g., after an accident or while on vacation.
- That I have full access to my records.
- I think it is simply timely and without alternatives.
- Intersectionality of the healthcare system is strengthened. Especially extremely useful for medication and emergency management (or if people are cognitively impaired).
- Central storage of all medical data simplifies keeping doctor's reports.
- Simplified data storage, digitization of processes
- As stated at the beginning, I currently do not use the ePA.
- None, because the proven insecure implementation (see CCC lectures on the subject) outweighs the benefits. I think age or a disability probably outweighs the advantages. As long as I can decide for myself, I can also look after my own data myself.
- You have everything at a glance and can view details of past treatments, sick notes, medications, etc. at any time at your leisure. This gives me the opportunity to better inform myself and also better understand what a doctor has documented. Especially in cases where you do not feel sufficiently informed or taken seriously, it is helpful to be able to review everything in writing afterward. This makes you feel safer.
- Access to my medications.
- All examination and treatment documents in one place. Past treatments can be more easily viewed by the doctor so that everything can be seen.
- Having an overview yourself, less paperwork at home.

- It is simple and efficient and would also be more sensible in the age of digitization.
- Easy access to all records in emergencies. An overview of all medications taken and possible interactions. Ensuring that no important information is lost.
- That it is digital.
- Overview of my medical history; no requests to doctors asking them to forward my data.
- The summary of medical matters is a great advantage for many people. It is also helpful for doctors to obtain more information about patients.
- Insight into diagnoses, health at a glance.
- Access to data in emergencies (knowingly). Automatic, uncomplicated, and complete transfer of data.
- Overview of medical history, insight into diagnoses and therapies.
- Overview of medications, diagnoses, findings in one place. Forwarding health data for research purposes. Easy access for medical staff to currently taken medications, pre-existing conditions, previous complaints, so that better-adapted treatment can be provided.
- All of my health information is bundled in one place and can be viewed, updated, and managed at any time without anything being lost.
- Central document storage. Reminders for vaccinations, preventive care, etc. Avoidance of duplicate or incorrect treatments.
- All medical documents centrally in one place, always available and easy to share with appropriate people.
- Central access.
- Organization and central management of all health information – selective sharing of data.
- Having all data in one place and being able to retrieve it at any time.
- In emergencies, I have everything under control.
- Better and easier communication between doctors.
- That no data can be lost.

Do you have concerns or barriers regarding the use of the ePA? If yes, which ones?

- Setup is somewhat time-consuming and requires some technical knowledge.
- I think doctors need to be very well trained in how to handle the ePA so that everything works as well as intended. For people who may not be very good with this technology, this would probably only lead to more problems.
- Since my general practitioner does not even look at the data that I myself entered during previous visits and often asks me the same questions several times, I doubt that they will take a close look at this record.
- I only know the ePA by name; I do not know much more about it.
- Not really. I just think that my GP practice will have major problems, as the practice is currently not even able to receive emails and is technically very outdated.
- An ePA can lead to medical staff relying only on what is written there and ignoring differential diagnoses. Especially in internal medicine and psychiatry, there are symptoms that can indicate multiple illnesses. I am also not sure whether entries on illnesses and medications also include the name of the treating person. It should be transparent who entered or deleted what and why.
- The app from my health insurance company crashes frequently or I cannot open it at all.
- Data leaks and misuse of my data, for example by supplementary insurance, such as disability insurance.
- Yes, selling my data, general sharing of data.
- I wonder how it is supposed to be ensured that no unauthorized access to patient data occurs. Everything that is stored electronically is, in my opinion, always exposed to the risk of external attacks, even if all necessary security measures are taken. The risk therefore always exists, even if it is small. In the event of such unauthorized access, these highly sensitive data could be used for purposes that are not in the patient's interest or, in the worst case, even against them. For example, if my data fell into the hands of a bank that denied me creditworthiness due to my health condition, that would probably not be an unlikely scenario and would leave many patients dissatisfied. I also wonder how these health data are anonymized at all and whether there are not manipulations in some form.
- Yes, I have concerns because I use the ePA very critically and deal with sensitive data, and I am not fully convinced that all important data are really protected against hacker attacks. Health data concern no one except me and my treating doctor, which explains my skepticism regarding the app's data security.
- I have no concerns. Since I have only gone to the doctor for routine check-ups for years and do not take any medication, there is currently no need.
- Data protection regarding the security of the infrastructure.
- Data protection and security.
- That the data could fall into the wrong hands or that it might appear difficult or confusing to me.
- Misuse by third parties.
- Negative effects on my insurance/benefits due to stored information.
- That access to data is limited and only people approved by me can access the data that I have approved.
- Trust in the use of the ePA naturally increases strongly with trust in the entire system. Political changes, for example, could also lead to increased mistrust regarding responsible handling of data.
- Fear that data will not (only) be used for non-profit research, but also or primarily misused for market or opinion research or commercially. Servers in Germany are also not exempt from hybrid warfare.
- Immature system, concerns regarding data protection and security.
- Due to the fact that online solutions often experience disruptions in many other areas, I think that a complete transition can only be implemented over time. Because in the event of a server crash, data that are important for individual patients could be deleted.
- Cyberattacks.
- No end-to-end encryption, various serious security problems (also from a system architecture perspective), consistent downplaying by those responsible, data protection being treated under the motto "AI will make everything great" without real understanding of the topic being apparent, pseudonymous data protection is not sufficient—anonymization would be required; see chat transcripts. I assume that this data is intended to be used not only for therapies but also for other purposes (see discussions with Microsoft and Google), opt-out instead of opt-in, potential future disadvantage for people without an ePA, societal stigmatization of people without an ePA.
- Lack of competence of Gematik regarding security. An app does not automatically make something better than a website.
- Yes, concerns about data leaks due to vulnerabilities in the technical implementation. Also concerns about whether known problems are noticed and fixed in a timely manner (see https://www.ccc.de/en/updates/2024/ende-der-epa-experimente). Finally, I am concerned whether access to the ePA will be implemented nationwide and promptly in medical practices.
- I do not want centralized storage of sensitive data. I would prefer local encrypted storage on my own device or card. I am also concerned that in the future not all data will be managed the way I want if, for example, health insurance companies benefit from it.
- In principle, I am positive about it. But there is always a risk that data will be misused. Security measures can never guarantee 100 percent protection. In this respect, I have slight concerns about what could happen if someone gains access to the data who should not have access.

- No system is 100 percent secure. Servers with sensitive medical data can be a lucrative target and would therefore be more likely to be attacked. In addition, what happens if the servers fail? Would my doctor be “blind” at that moment?
- The ePA stores personal data outside of my control.
  The data are more easily accessible to third parties due to centralization.
  Access rights management is not implemented selectively enough.
  Major security gaps are known to me and are openly acknowledged.
- Yes, nowadays you can’t trust anyone.
- Data protection, security of stored data against third parties.
- The app is very cumbersome, partly due to (reasonable) security precautions—the setup and identification process was generally very time-consuming. It is also questionable whether the ePA could lead to increased workload for medical practices—time that would then be missing for patients.
- Regarding data, it is also partly common that data are sold to other countries or on the black market.
- I doubt the security of the ePA. The personal data of an extremely large number of people in one place could become a target for cybercrime.
- In the long term, the data could theoretically be used to identify me as a high-risk patient or similar and calculate different contributions; misuse; data leaks.
- I am concerned that diagnoses, which are not always correct, could lead doctors and healthcare staff to draw incorrect conclusions.
- Leaks, hackers.
- Data security has not yet been guaranteed (which is also delaying implementation).
  Centralized storage is always associated with risks of data misuse.
- Technology is also not error-free. Data leaks, short circuits where data are not stored, possibly due to Wi-Fi connections.
- Lengthy registration process; little education about data protection.
- As long as everything is developed according to the latest security standards and safeguarded through adequate backup systems, I have no concerns.
- Lack of transparency – too much uncertainty.
- No, I think it is time for such a technology, and no technology is perfect at release.
- My concern is that it may fail due to insufficient training of all ePA stakeholders or that our “never change a running system” mentality will create a double burden for a certain period of time. It would be good to keep the adjustment period as short as possible so that as little chaos as possible arises.
